# Multi-scale, whole-system models of liver metabolic adaptation to fat and sugar in non-alcoholic fatty liver disease

**DOI:** 10.1038/s41540-018-0070-3

**Published:** 2018-08-20

**Authors:** Elaina M. Maldonado, Ciarán P. Fisher, Dawn J. Mazzatti, Amy L. Barber, Marcus J. Tindall, Nicholas J. Plant, Andrzej M. Kierzek, J. Bernadette Moore

**Affiliations:** 1 School of Biosciences and Medicine, University of Surrey, Guildford, Surrey, GU2 7XH UK; 2 Certara UK Limited, Simcyp Division, Level 2-Acero, 1 Concourse Way, Sheffield, S1 2BJ UK; 3 Proctor & Gamble, Cincinnati, OH 45224 USA; 4 Department of Mathematics and Statistics, University of Reading, Berkshire, RG6 6AX UK; 5 Institute of Cardiovascular and Metabolic Research, University of Reading, Berkshire, RG6 6UR UK; 6 Faculty of Biological Sciences, University of Leeds, Leeds, West Yorkshire, LS2 9JT UK; 7 School of Food Science & Nutrition, University of Leeds, Leeds, West Yorkshire, LS2 9JT UK

## Abstract

Non-alcoholic fatty liver disease (NAFLD) is a serious public health issue associated with high fat, high sugar diets. However, the molecular mechanisms mediating NAFLD pathogenesis are only partially understood. Here we adopt an iterative multi-scale, systems biology approach coupled to in vitro experimentation to investigate the roles of sugar and fat metabolism in NAFLD pathogenesis. The use of fructose as a sweetening agent is controversial; to explore this, we developed a predictive model of human monosaccharide transport, signalling and metabolism. The resulting quantitative model comprising a kinetic model describing monosaccharide transport and insulin signalling integrated with a hepatocyte-specific genome-scale metabolic network (GSMN). Differential kinetics for the utilisation of glucose and fructose were predicted, but the resultant triacylglycerol production was predicted to be similar for monosaccharides; these predictions were verified by in vitro data. The role of physiological adaptation to lipid overload was explored through the comprehensive reconstruction of the peroxisome proliferator activated receptor alpha (PPARα) regulome integrated with a hepatocyte-specific GSMN. The resulting qualitative model reproduced metabolic responses to increased fatty acid levels and mimicked lipid loading in vitro. The model predicted that activation of PPARα by lipids produces a biphasic response, which initially exacerbates steatosis. Our data support the evidence that it is the quantity of sugar rather than the type that is critical in driving the steatotic response. Furthermore, we predict PPARα-mediated adaptations to hepatic lipid overload, shedding light on potential challenges for the use of PPARα agonists to treat NAFLD.

## Fatty liver disease: Liver adaptation to sugars and fats

Novel computational modelling approaches predict the liver’s metabolic response to dietary fat and sugar. A multi-disciplinary team led by Dr. J. Bernadette Moore at the University of Leeds has used novel computational modelling and simulation approaches to understand the livers response to dietary sugars and fats, and the development of non-alcoholic fatty liver disease. Simulations of the team’s multi-scale models, along with their experimental findings, show it is the quantity, not type (e.g. fructose vs. glucose), of sugar that drives fat accumulation in liver cells. Furthermore, they model the behaviour of PPARα, a critical regulator of fat metabolism in the liver, providing cautionary insights into its potential as a therapeutic target. This work demonstrates the potential of systems approaches to elucidate mechanisms underpinning the role of diet in non-alcoholic fatty liver disease.

## Introduction

Non-alcoholic fatty liver (NAFLD) disease is a major public health concern associated with obesity and the metabolic syndrome. Prevalence is estimated at 30–45% of the adult population in many countries, although NAFLD is typically underdiagnosed due to its asymptomatic nature in its initial stages. Pathogenesis begins with steatosis, the accumulation of lipid droplets within the hepatocytes of the liver. Although steatosis can be reversible and may be viewed as relatively benign clinically,^1^ it has long been a recognised marker of liver damage that is known to alter the metabolism and disposition of therapeutic drugs due to alterations in activity of metabolising enzymes.^2^ Moreover, steatosis can progress to non-alcoholic steatohepatitis (NASH), which involves a series of inflammatory responses in the liver. While still potentially reversible, NASH is associated with increased incidence of fibrosis, hepatocellular carcinoma, liver failure and the need for liver transplant.^1^ Despite the high prevalence of this disease, particularly in obese individuals, the pathogenesis of NAFLD is complex and not well understood, limiting the development of effective treatments. Currently, there are no pharmaceutical agents licenced for the treatment of NAFLD with weight loss, dietary and lifestyle modifications underpinning clinical management.^3^ Whether or not a low-sugar or low-fat diet should be recommended for NAFLD is controversial and we consider these dietary factors here.

Dietary sugars, in particular fructose, have been implicated in the development and progression of NAFLD and other chronic metabolic diseases.^4^ Fructose has been scrutinised in part due to its extensive use in beverages such as fizzy and fruit-flavoured drinks for which children and adolescents are major consumers. High fructose intakes have been shown to alter hepatic insulin sensitivity, increase lipogenesis and ectopic lipid disposition in human and rodent studies.^5^ Hepatic fructose metabolism bypasses a key rate-limiting step in glycolysis leading to the provision of increased substrates for de novo lipogenesis (DNL) and the increased synthesis of long chain fatty acids, triacylglycerol (TAG) and other, often inflammatory, lipid intermediates.^6^ In addition, fructose regulates the activity of multiple transcription factors involved in the regulation of both lipogenesis and fatty acid oxidation including the carbohydrate response element binding protein (ChREBP), the sterol response element binding protein (SREBP1) and the peroxisome proliferator activated receptor alpha (PPARα).^5,7^ However, the impact of fructose at lower, ‘normal consumer’ levels is debatable, and it is unclear if there is a differential impact on lipogenesis and NAFLD beyond the provision of, typically, excess energy.

Recent focus on the negative metabolic effects of a high-sugar diet has led to debate over historical dietary guidelines, which recommend low-fat (considered <35% of daily energy from fat with an ‘acceptable distribution’ of 20–35%) and low-saturated fat diets (7–10% of total energy) for the prevention of cardiovascular disease.^8,9^ The critical point frequently neglected in the, often polarised, debates around whether sugar or fat is the nutrition villain of the day,^10,11^ is the key observation that, at a population level, identifying individual culpable nutrients is problematic. While almost no one consumes a low-fat diet (US^12^ and UK^13^ adults consume an average of 34–35% of daily energy intake from fat), the vast majority of adults in developed countries consume excess energy from foods high in both sugar and fat, fundamentally contributing to increasing obesity and NAFLD.

While it is generally accepted that high-sugar and high-fat diets disrupt metabolic homoeostasis and the regulation of lipogenesis, thereby contributing to NAFLD, the molecular mechanisms and temporal order of key metabolic and signalling events are unclear. Here in silico models and systems biology approaches may offer insights into disease mechanisms, generating hypotheses for subsequent experimental testing.^14^ Computational systems biology has advanced considerably in the last decade, including the reconstruction of human genome-scale metabolic networks (GSMNs), comprehensive models incorporating the metabolic and transport reactions mediated by the proteins encoded within the genome.^15–17^ These models can be further constrained to reflect specific tissues, or even specific organelles, using transcriptomic data.^18–20^ Recently GSMNs have been utilised for the mechanistic interpretation of clinical NAFLD data.^21,22^ However, these models are static and do not reflect the dynamic reprogramming of global metabolism and metabolic adaptation to maintain homoeostasis during sugar and lipid loading. Increasingly, GSMNs are being integrated with detailed gene regulatory and/or physiologically based pharmaco/toxico-kinetic models in the emergent fields of quantitative systems pharmacology/toxicology.^23^

Here we adopt a multi-scale approach, coupling GSMNs to dynamic models of regulatory networks^23–25^ to study how disruption of metabolic adaptation contributes to NAFLD pathogenesis. We examine two important questions in NAFLD; the differential impact of glucose and fructose on lipogenesis, and the role of the PPARα regulome in liver adaptation to lipid loading.

## Results

Using the systems approach outlined in Fig. S1, two questions related to NAFLD were explored. First, we examined an area of debate in the NAFLD literature;^4,5^ specifically, whether or not fructose is more lipogenic than glucose. Utilising the QSSPN^24^ method, we reconstructed a dynamic, quantitative model of monosaccharide transport and fully parameterised insulin signalling;^26^ and integrated this to a hepatocyte-specific GSMN^19^ with an external nutrient exchange set^27^ representing in vitro conditions constrained by in vitro consumption and secretion rates (see Supplementary Tables S1-S4 and Fig S2-S3). Alongside this computational approach, monosaccharide and insulin-treated hepatocytes (HepG2 cells) were utilised for in vitro experiments to aid in model validation and hypothesis testing.

Secondly, we conducted a transcriptomics study to identify pathways perturbed by lipid loading in immortalised human hepatocytes. The data-driven, pathway enrichment analysis of this data, as well as additional proteomic datasets from our preclinical and in vitro models of NAFLD, identified the PPARα regulome as a key regulatory network module in liver adaptation to lipid loading.^28,29^ Due to the large scale of this omics data derived regulatory network, parameterisation of a fully quantitative model was not feasible. Therefore, we capitalised on the flexibility of the multi-formalism QSSPN method and developed a qualitative dynamic model, again coupled to the hepatic GSMN. In both cases, model simulations drove experimental design and further model refinement in an iterative fashion, generating hypotheses that were tested in vitro.

### Multi-scale modelling of hepatic monosaccharide metabolism

#### In silico model recapitulates in vitro sugar transport

**Fig. 1:**
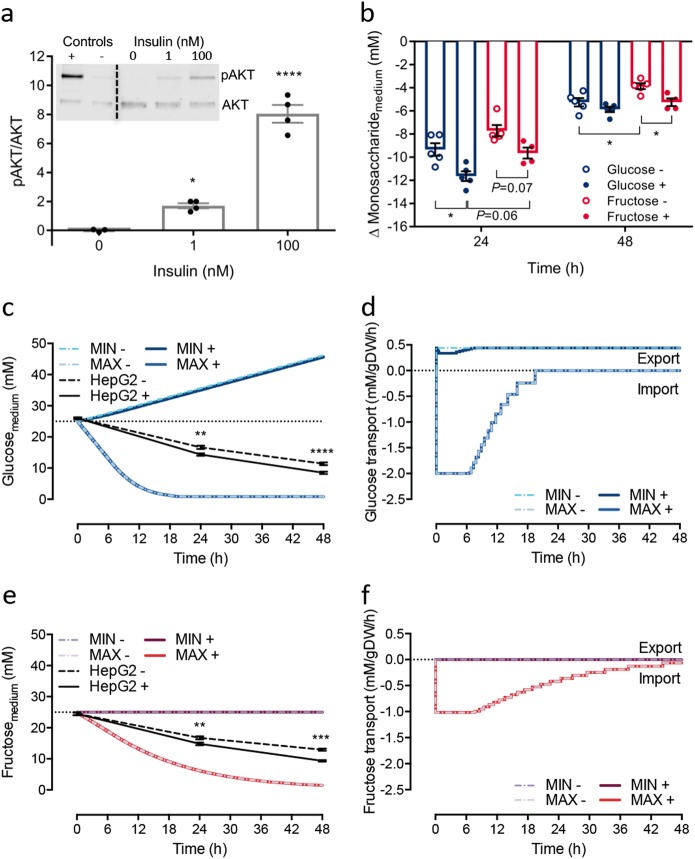
Insulin sensitivity and verification of sugar consumption in vitro and in silico. **a** Immunoblot analyses of pAKT/AKT expression (both ~60 kDa) in HepG2 cells stimulated with insulin (*n* = 3–4), analysed by one-way ANOVA with Dunnett’s test post hoc between doses and vehicle. **b** The change in monosaccharide concentration of culture medium in vitro over the first and second 24 h period after treatments of glucose or fructose with (+) and without (−) 100 nM insulin (*n* = 4–5), analysed within timepoints between treatment by one-way ANOVA with Tukey’s test post hoc. **c**–**f** The objective function was set as either the glucose or fructose transport flux between the external space (medium) and sinusoid space. Maximisation was the uptake of monosaccharide, and minimisation was the production and export into the medium (external space). **c** Model predictions of glucose concentration in the medium with (+) or without (−) the presence of 100 nM insulin over time alongside experimental data from HepG2 cells (*n* = 3–5). **d** Predicted glucose transport rate over time. **e** Predictions of fructose concentration over time alongside experimental data from HepG2 cells (*n* = 3–5). **f** Predicted fructose transport rate over time. Data shown as mean ± SEM. Statistical differences are indicated as * *P* < 0.05, ** *P* < 0.01, *** *P* < 0.001, and **** *P* < 0.0001

Human hepatoma (HepG2) cells were first treated with physiological and supraphysiological concentrations of insulin and sugars to mimic the human hepatic overfed state in vivo. We confirmed the responsiveness of HepG2 cells to insulin stimulation by measuring the phosphorylation ratio of the RAC-alpha serine/threonine-protein kinase (pAKT/AKT) protein, which transduces signalling from the insulin receptor. In response to a postprandial-like dose of 1 nM insulin, a 1.7-fold increase in pAKT/AKT (*P* < 0.05) was observed (Fig. 1a), while 100 nM of insulin elicited an 8-fold increase in pAKT/AKT (*P* < 0.0001; Fig. 1a). Insulin treatment (100 nM) increased the rate of monosaccharide disappearance from the medium relative to non-insulin stimulated cells (Fig. 1b). HepG2 cells consumed glucose at a faster rate than fructose (Fig. 1b); after the first 24 h, glucose concentrations in medium decreased by 9.3 ± 0.55 mM (equivalent to 0.97 mg/million cells) and 11.6 ± 0.42 mM (1.14 mg/million cells) in non-insulin and insulin stimulated cells, respectively. Meanwhile, fructose decreased by 7.7 ± 0.49 mM (0.82 mg/million cells) and 9.6 ± 0.48 mM (1.0 mg/million cells) in non-insulin and insulin-treated cells, respectively. During the second 24-h period, the average uptake of glucose was still more than the uptake of fructose (as measured by clearance from medium), with a decrease in concentration of glucose by 5.2 ± 0.36 mM (0.44 mg/million cells) and 5.9 ± 0.23 mM (0.47 mg/million cells) and of fructose by 3.9 ± 0.25 mM (0.39 mg/million cells) and 5.2 ± 0.34 mM (0.49 mg/million cells) in non-insulin and insulin treated cells, respectively. At this time point, insulin still caused a significant increase in fructose uptake (*P* < 0.05), whereas the effect of insulin on glucose uptake disappeared (Fig. 1b).

To validate the in silico model with experimental data on monosaccharide uptake, initial conditions were set with or without 100 nM of insulin with either 25 mM of glucose (Fig. 1c) or fructose (Fig. 1e). We note that model parameters were not adjusted to improve the fit of the model to experimental data, allowing evaluation of the predictive power of the model. To explore the predicted range of sugar utilisation, we formulated a dynamic flux variability analysis (dFVA) simulation protocol (described in detail in Supplementary Methods), with maximisation of the objective function (i.e. glucose transport, reaction ID: EX_Glucose; and fructose transport, reaction ID: EX_Fructose) representing consumption, and minimisation representing production. To the best of our knowledge, this is the first application of dFVA to a quasi-steady state, dynamic simulation.

QSSPN dFVA maximisation of glucose transport predicts a rapid depletion of glucose from medium (Fig. 1c), which is complete after 20 h. Minimisation of glucose transport predicts an accumulation of glucose in the medium due to gluconeogenesis. Likewise, maximisation of fructose transport resulted in depletion of medium fructose, although this was slower than predicted for glucose and not fully complete within the 48-h simulation period. As expected, no significant accumulation of fructose in medium was predicted for minimisation of transport (Fig. 1e). Acute insulin stimulation of HepG2 cells resulted in an increased rate of depletion of both glucose and fructose from medium (Fig. 1c, e). dFVA minimisation of glucose transport predicts a small decrease in the rate of accumulation in the medium in the presence of insulin. This effect can be more clearly seen by examining the rate of glucose flux (Fig. 1d); production and export of glucose was initially reduced, recovering to baseline within 6 h. In contrast, no impact on fructose transport was predicted with insulin stimulation (Fig. 1f). This may be due to the features of dFVA, which predicts the absolute minimal and maximal reaction rate to provide a unique feasible solution space given the conditions of the computational model. Nonetheless in both instances, the in vitro data were found to fit well within the predicted dFVA solution space.

#### De novo intracellular lipid in silico and in vitro

**Fig. 2:**
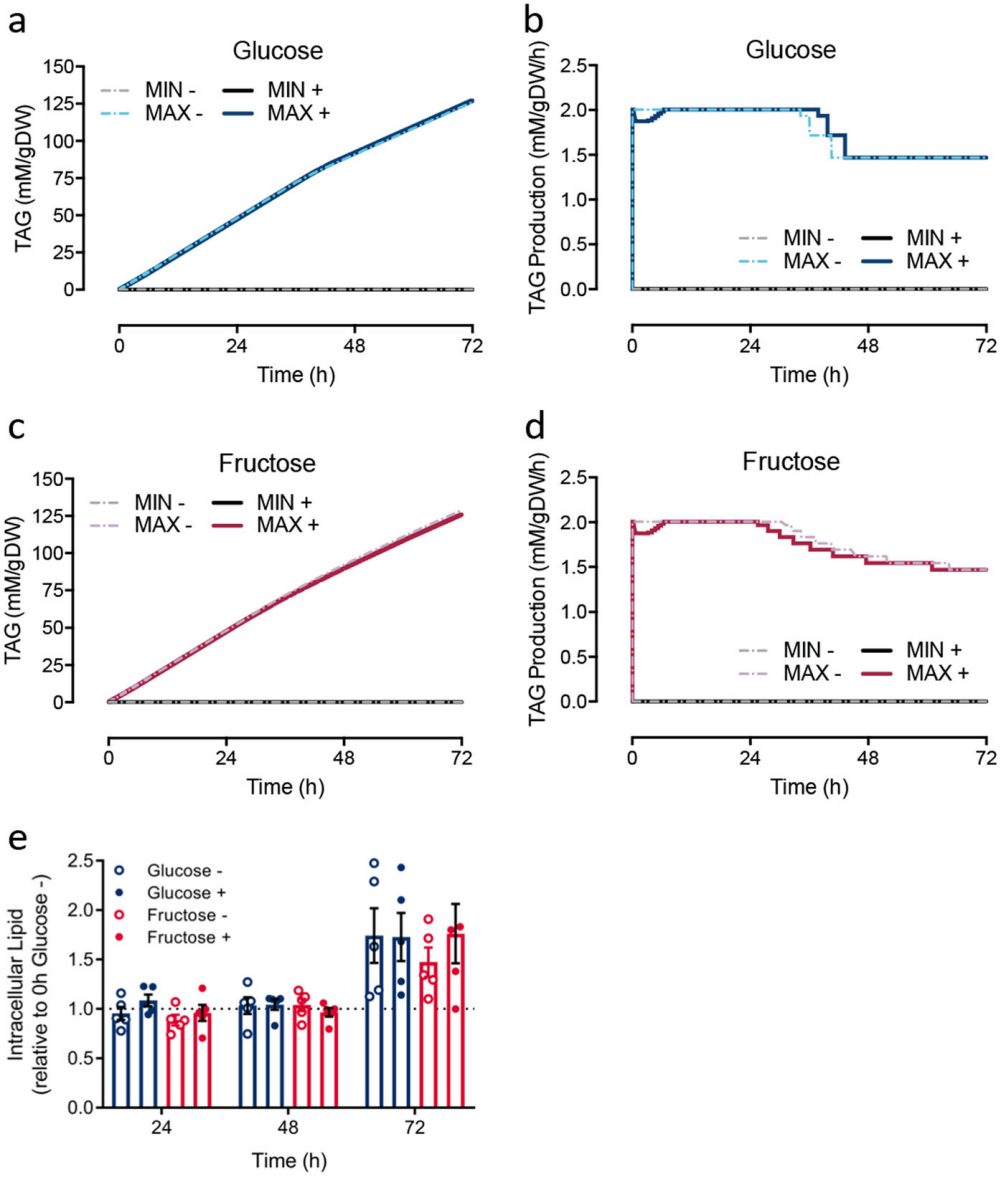
Predicted intracellular triacylglycerol (TAG) in silico and intracellular lipid measured in vitro. **a**–**d** The objective function was set as TAG production within the cytosol. Maximisation was the maximum production of TAG and minimisation was towards null production. Representing the cell experiment, the model initial state was set with either 25 mM glucose or fructose with (+) or without (−) the presence of 100 nM insulin. **a** Predicted intracellular TAG concentrations from glucose. **b** Predicted TAG production from glucose over time. **c** Predicted intracellular TAG concentrations from fructose. **d** Predicted TAG production from fructose over time. **e** Intracellular lipid in HepG2 cells (*n* = 5) measured by Nile red staining at every 24 h for 72 h. Media were not replenished during the period of measurements. Data shown as mean ± SEM, adjusted to background fluorescence from non-Nile red stained cells, and expressed relative to 25 mM glucose without insulin treatment at 0 h. Two-way ANOVA with Tukey’s test post hoc was performed. No differences were detected between treatments

The accumulation of lipid in macrovesicular droplets is the hallmark of steatosis. It is therefore important to explore not only the ability of hepatocytes to utilise sugars, but also their subsequent conversion into lipid. As triacylglycerol (TAG) is the predominate lipid species within the neutral lipid core of macrovesicular droplets,^30^ the in silico model was then used to investigate the influence of monosaccharide type on TAG metabolism. TAG production (reaction ID: r1223) was set as the objective function and dFVA was used to explore the maximal and minimal TAG production possible for a given monosaccharide. With an initial concentration of 25 mM glucose, maximisation of TAG production resulted in a rapid production of TAG over time (2.01 mM/g DW/h; Fig. 2a). This initial rate decreased to 1.47 mM/g DW/h after ~40 h, as a result of glucose depletion from the system (Fig. 2b). With fructose as a substrate, the maximised initial TAG production rate (2.01 mM/g DW/h) was no different from simulations with glucose (Fig. 2c). Likewise, a decrease in TAG production rate occurred, although this was apparent at an earlier time point (approximately 24 h; Fig. 2d). The area under the time-concentration curve (AUC_0-48_) for TAG was 2300 and 2332 mM.h/g DW for glucose (48.2 h) and fructose (48.5 h), respectively. As expected, minimisation of TAG production resulted in no production of TAG, regardless of monosaccharide (Fig. 2a, c). The simulation of insulin treatment predicted an initial adjustment of the metabolic network landscape before stabilising at the maximum TAG production rate of 2.01 mM/g DW/h by 6.4 h (Fig. 2b). Insulin treatment lead to only a subtle decrease in overall TAG production, with predicted AUC_0-48_ values of 2288 and 2281 mM.h/g DW for glucose and fructose, respectively.

To confirm the in silico predictions, HepG2 cells were used to detect the influence of the type of monosaccharide, with and without insulin, on hepatic intracellular lipid in vitro. HepG2 cells, with or without 100 nM of insulin, were exposed to either 25 mM of glucose or fructose and measured every 24 h over 72 h by Nile red staining (Fig. 2e). Consistent with the in silico prediction, lipid accumulation was found to be no different when glucose or fructose was used as a carbon source. In addition, insulin stimulation had no significant impact on intracellular lipid levels expressed as relative to 25 mM glucose treated cells without insulin stimulation at 0 h. This is consistent with the in silico prediction of no long-term differences in TAG production after insulin stimulation for either glucose or fructose.

### Impact of the PPARα regulome on hepatocyte lipid loading

**Fig. 3:**
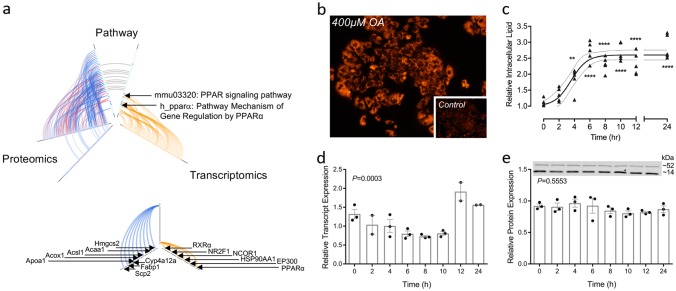
PPARα mRNA and protein expression in in vivo and in vitro models of NAFLD. **a** Hive plot summarising pathway enrichment analysis against the KEGG and BIOCARTA databases ranked by clustering coefficient. Proteomics identified 31 enriched pathways (Alzheimer’s and Parkinson’s excluded from analysis), 11 pathways identified in both the membrane and cytosolic fractions, only 15 mapped proteins identified in both the membrane (blue) and cytosolic (red) fractions. Transcriptomics (orange) identified 10 enriched pathways; smaller plot identifies transcripts and proteins contributing to the enrichment of PPARα protein. **b** Fluorescence micrograph (×100) of HepG2 cells treated for 24 h with 400 μM oleate; control cells inset; **c** relative intracellular lipid quantified by Nile red fluorescence mean ± SEM (*n* = 5) data analysed using one-way ANOVA with Tukey’s test post hoc, **P < 0.01, ****P < 0.0001 vs. 0 h timepoint; **d** relative expression of PPARα transcript determined by qRT-PCR mean ± SEM (*n* = 3), data analysed using one-way ANOVA; **e** quantification of relative PPARα protein expression mean ± SEM (*n* = 2-3; representative western blot shown), data analysed using one-way ANOVA

We have previously reported on transcriptomic and proteomic analyses in preclinical models of NAFLD; including fatty acid-treated hepatocytes (mimicking steatosis or NASH depending on fatty acid composition used), and high-fat fed murine models.^28,31^ The prior identification of disruption and activation of PPARα signalling in pilot experiments in palmitate-treated hepatocytes (HuH7 cells),^28^ was confirmed independently here in a combined pathway enrichment analysis of both the proteomics^31^ and microarray data sets.^28^ Enriched pathways identified as statistically significant (*P* < 0.05) were ranked by clustering coefficient and presented with results from both datasets using hive plots^32^ (Fig. 3a). Enriched PPARα pathways were identified independently in both datasets against multiple databases (KEGG and BIOCARTA).

With these data as our rationale, a PPARα regulome reconstruction was manually curated from a thorough search of the peer-reviewed literature and online databases (Fig S1). The final model dynamically regulates the expression of 91 PPARα-target genes, modulating flux through 233 metabolic and transport processes; full details are presented in Supplementary Methods. Since there is still insufficient data available to quantitatively parameterise such a large regulatory network model, the QSSPN approach was used to create a comprehensive qualitative model of the PPARα regulome integrated with the hepatocyte-specific GSMN, HepatoNet1.^19^ This allowed examination of the dynamic cellular response behaviours to lipid loading. Although previous reconstructions of the PPARα regulatory network have been published,^33^ we believe our resulting model to be the most comprehensive model of PPARα-regulated metabolism described to date.

#### Lipid loading and PPARα expression in vitro

PPARα mRNA transcript and protein levels were measured in HepG2 cells treated with 400 μM oleic acid (OA) over 24 h (Fig. 3b–e). Intracellular lipid accumulation was monitored through quantification of Nile red fluorescence (Fig. 3b, c) at intervals of 2 h for the first 12 h and then at 24 h, with RNA and protein collected at each time point (Fig. 3d, e). Intracellular lipid increased in response to OA treatment, reaching a maximum after 6 h (Fig. 3c) and resulted in clear macrovesicular steatosis under fluorescent microscopy by 24 h (Fig. 3b). PPARα transcript levels exhibited a significant change in relative levels over time (one-way ANOVA, *P* = 0.0003; Fig. 3c, d); however, this did not translate to a significant change in relative protein expression (Fig. 3e).

#### QSSPN simulation of PPARα-regulated metabolism

Building on previous work,^24^ the gene regulatory model we employ here is rule-based and has three stable levels of gene expression, allowing for genes expressed at basal steady-state to be reversibly induced or inhibited (Fig. S4). The reconstructed QSSPN model was used to simulate a dynamic response to lipid loading. Following the qualitative simulation approach described before,^24^ we performed Monte Carlo simulations to generate multiple dynamic trajectories representing sequences of feasible molecular events given rules defining regulatory networks and the stoichiometric constraints of the metabolic model. Simulation time was in arbitrary units and reflected exclusively the order of events. The maximal length of the simulation was set so that simulation time courses covered the whole range of homoeostatic response, allowing observation of the return to baseline.

Changes in metabolic flux from a representative simulated trajectory were illustrated in an adjacency matrix heatmap capturing the reconstructed PPARα regulome and regulated fluxes within the GSMN (Fig. S5). This maps 91 PPARα target genes and the 233 metabolic/transport reactions within the GSMN; these are detailed in supplementary Table S7. After fatty acid stimulation, reaction fluxes are predicted to rapidly alter, both in terms of magnitude and directionality, as part of an acute regulatory response. Alterations in fluxes during the acute regulatory phase are attributable to the sequential, and within this simulation, stochastic reprogramming of protein expression through PPARα-mediated regulation. Subsequent to this, the system reverts to the baseline over time (reconstitutive phase). Within the reconstitutive phase, we note that persistent differential flux values persist after fatty acid treatment has been cleared, indicating an extended regulatory effect following clearance of the agonist (i.e. a regulatory ripple effect). Simulations with a regulatory naive model (i.e. no PPARα-mediated regulation) showed no such reconstitutive phase and far fewer altered fluxes in response to fatty acid treatment (Fig. S6). This highlights the importance of transcription factors, in particular PPARα, in coordinating adaptive response to lipid loading in hepatocytes.

#### PPARα activation in silico and in vitro

**Fig. 4:**
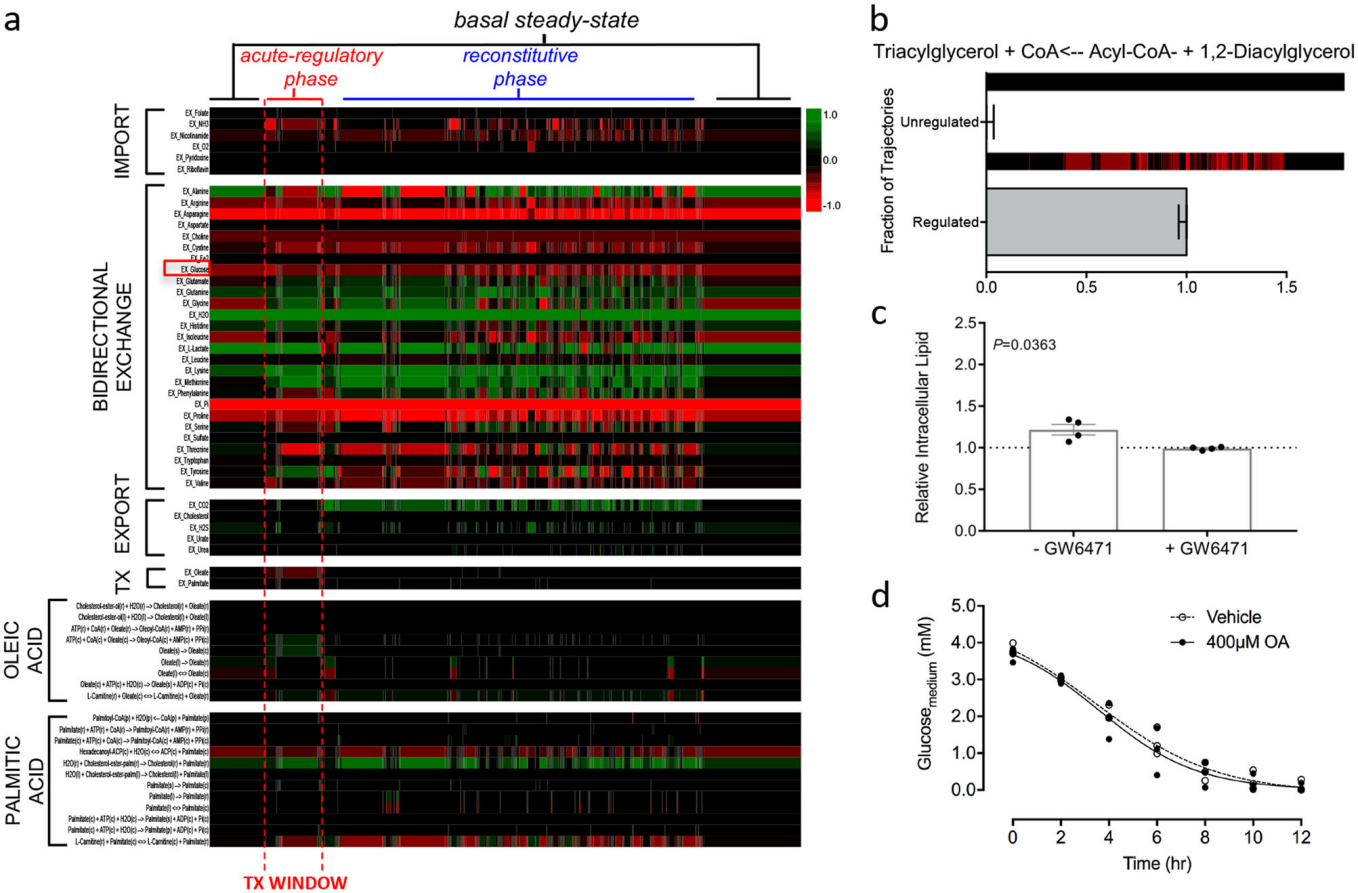
Indirect effects of PPARα-mediated metabolic adaption. **a** Single simulated trajectory heatmap of exchange set fluxes, treatment fluxes and all fluxes where palmitate and oleate are primary metabolites or products. Positive flux values are shown in green, negative flux values in red with simulated time progressing left to right. Glucose bidirectional exchange highlighted with red square. **b** Fraction of trajectories showing increased flux towards TAG synthesis sampling 100 trajectories with single representative trajectory of TAG flux shown above as heat map, data shown as fraction of trajectories ± binomial probability confidence intervals. **c** Relative intracellular lipid as quantified by Nile red fluorescence in HepG2 cells treated with 400 μM oleic acid for 2 h ± PPARα antagonist GW6471 mean ± SEM (*n* = 4) relative to vehicle and analysed using a two-tailed t-test with Welch’s correction. **d** Glucose concentrations in the culture media of HepG2 cells treated with vehicle or 400 μM oleic acid over mean ± SEM (*n* = 3)

To further explore the biological impact of PPARα-mediated gene regulation in response to lipid loading, we monitored metabolic fluxes towards TAG as a representative endpoint in steatosis (Fig. 4a). We examined fluxes through reactions involved in nutrient transport and fatty acid metabolism, allowing an exploration of the wider, indirect effects of the PPARα regulome on hepatocyte metabolism. Large alterations in reaction fluxes were predicted in exchange reaction fluxes that represent nutrient supply from the cell exterior, specifically those associated with amino acid transport. Simulations with the regulatory incompetent model predicted no increased flux towards TAG in response to simulated treatment with OA (Fig. 4b), consistent with the requirement for PPARα regulation to support TAG production. This indicates that hepatic steatosis is an emergent property of metabolic regulation, rather than just a simple result of increased flux through the metabolic network in response to the increased availability of substrate. Furthermore, and possibly contrary to putative understanding, PPARα activation by intracellular fatty acids would appear to result in the increased production of TAG rather than clearing accumulated lipid.

Using the in vitro HepG2 steatosis model, we proceeded to test this hypothesis. Using the PPARα antagonist GW6471, we repeated our previous experiments, treating cells with 400 μM OA in the presence or absence of the antagonist. Consistent with the in silico prediction, we observed a statistically significant decrease in the level of lipid accumulated after 2 h of treatment with OA and GW6471 compared with controls (Fig. 4c). After 24 h, no difference in the level of lipid accumulation was seen with or without GW6471 (Fig. S7). Thus, the acute regulatory response in vitro can be inhibited through use of a competitive PPARα inhibitor, however, this protective effect appears to be over-whelmed within 24 h indicating the role of further regulatory mechanisms to which the in silico model is currently naïve. Complimentary to our investigations in to the role of carbohydrates in hepatic steatosis, we monitored the consumption of glucose over the first 12 h of treatment with OA. The accumulation of lipid within the cells has no statistically significant impact on the rate of glucose consumption (Fig. 4d). This is consistent with our simulated results that show minimal changes in glucose consumption flux values over course of simulations (Fig. 4a, red box highlights glucose bidirectional exchange).

Figure 4a (and Figure S5) illustrates an important characteristic of the regulatory process; the biological response is not limited to the duration of the signal and can be described as multi-phasic. The acute regulatory phase is the immediate response seen in the presence of agonist-activated PPARα. Here, PPARα-mediated changes in gene expression alter the metabolic landscape of the cell, resulting in an altered flux distribution. On loss of the agonist, the system does not immediately return to the basal state. Instead the system goes through a reconstitutive phase that extends beyond the presence of the signal. This extended reconstitutive phase may represent the decay time of produced proteins, alterations in reaction fluxes to maintain cellular homoeostasis under varying conditions, or a combination of the two. It should also be noted that both the acute and reconstitutive phases of regulation are not simply limited to fluxes directly linked to the PPARα regulome (Fig. S5) but propagate through the metabolic network causing indirect regulation (Fig. 4a). This represents the impact of altered levels of substrates and/or co-factors on the metabolic landscape; for example, a reaction may be limited by the depletion of a co-factor utilised by another reaction. The simulation results presented here are not on a quantitative time scale yet permit the exploration of the order in which events occur, providing important insight into the design principles of the biological system. For example, we can hypothesise that if the PPARα regulome is activated at intervals such that the system never completes the reconstitutive phase, then the metabolic landscape will not be able to return to its basal steady-state. The pathogenesis of metabolic disorders such as NAFLD may be a resultant effect of such persistent disruptions observed.

## Discussion

Associated with the exponential rise in obesity, NAFLD has quickly become the most common chronic liver disease in many countries. Often undetected for many years, NAFLD increases the risk of chronic diseases such as type 2 diabetes and cardiovascular disease, and alters drug metabolism in the liver.^2^ While it is evident that NAFLD pathogenesis involves altered hepatic lipid metabolism, likely stemming from a combination of environmental insults (e.g. over nutrition) and genetic susceptibility, the underlying mechanisms remain unresolved. This lack of understanding has limited the ability to predict progression along the NAFLD spectrum for an individual, or to design effective therapeutic interventions and/or dietary advice. With this motivation, in this work we address two of the outstanding questions within the NAFLD field: does fructose, commonly used as a sweetener, have a greater lipogenic potential than glucose; and, what is the biological impact of activation of PPARα by fatty acids during NAFLD? To address these questions, we have reconstructed hepatic regulatory models for both monosaccharide signalling and transport and the PPARα regulatory network, then integrated these to a genome scale model of hepatic metabolism utilising QSSPN.

The potential differential effects of glucose and fructose on TAG metabolism were investigated with a reconstruction comprising monosaccharide transport, insulin signalling and hepatic metabolism. The resulting multi-scale, hybrid model was used to perform predictive, dynamic simulations of minimum and maximum flux rates. No differential lipogenic effects were predicted between fructose and glucose, a result verified by experiments in HepG2 cells. These results are important in the context of ongoing disagreement in the literature about potential differential effects of fructose on health and NAFLD.^34^ Studies using supra-physiological doses of single monosaccharides suggest some differential effects, but are difficult to reconcile with the diets of free-living humans. In addition, meta-analyses of studies with blood lipid and liver-related end points show no effect in trials that controlled for energy.^35–37^ Studies in vitro also present conflicting conclusions: some studies showing differential effects of fructose at remarkably low (0.72 mM) concentrations of total monosaccharide with increasing fructose:glucose molar ratios;^38^ other studies show no differential effects until higher (25 mM) doses of fructose were used.^39^ Such concentrations are generally supra-physiological doses, being significantly higher than concentrations observed in portal blood, which rarely exceed 2 mM. As recently shown by Jang and colleagues (2018),^40^ the majority of fructose is metabolised in the intestine with extensive fructose-derived glucose, lactate and glycerate found in portal circulation. While we did not simulate this metabolic interconversion in our kinetic model, such metabolic fates are captured within the hepatic model. We did not explore if these metabolic fates were realised in silico by tracing the full metabolic fate of fructose. An interesting future direction would be to expose the cell lines to this mixture of metabolites and compare the response to when the parent (fructose) is given. Additionally, in studies where very small positive effects of fructose on intracellular lipid in HepG2 cells have been shown,^41,42^ there is uncertainty about the minimal limit of detection of the lipid assay used.

Supporting our results are data from a recently published kinetic model comprising 88 reactions and 81 metabolites of hepatocyte core metabolism capable of simulating energy and redox metabolism.^43^ The model of Fouget and colleagues was parametrised with ^13^C labelled glucose and fructose based experimental (GC-MS) data from primary rat hepatocytes treated with 20 mM of glucose supplemented with either 3 or 20 mM of fructose for 2 h. The results show that while 20 mM of fructose inhibited glycogen synthesis, the addition of 3 mM of fructose, a physiological relevant portal concentration (0.2–2 mM^44^), showed no adverse effects on intracellular energy status. Moreover, the authors observe there was very little flux going through fatty acid synthesis in both experimental models of 3 and 20 mM fructose. The authors attributed this outcome to isolating primary hepatocytes from fasted rats, plus the short experimental incubation time. While the in vitro experiments performed here did not take into account glycogen synthesis; similarly, our experimental HepG2 cells fed glucose and fructose had no differential effects in intracellular lipid over longer periods of exposure. It might be of interest to implement the stoichiometric data produced from Foguet and colleagues^43^ into our metabolic model in order to incorporate specific rates of hepatic fructose and glucose metabolism. However, whereas our data are from a human hepatoma cell line, the Foguet data are derived from rat primary hepatocytes; ultimately human kinetic data would be preferable. In summary, through the use of a biologically realistic computational model encompassing the key mechanistic modules of monosaccharide processing, we conclude that there is no differential effect on lipogenesis between glucose and fructose.

The identification of PPARα as a key regulatory network module in liver adaptation to lipid loading was interesting given the potential clinical role for PPARα activation in NAFLD treatment, with dual and selective PPAR modulators currently in phase 2 and phase 3 clinical trials.^45^ Activation of PPARα induces genes involved in fatty acid binding, transport, and β-oxidation, thereby promoting the uptake, utilisation, and catabolism of fatty acids. In NAFLD patients, liver PPARα gene expression negatively correlates with NASH severity, visceral adiposity and insulin resistance.^46^ The serum lipid lowering fibrates are established weak agonists of PPARα used to treat atherogenic dyslipidaemia. However, fibrates have had disappointing results in trials examining their use in NAFLD, with no improvement in histological NASH observed in several pilot studies.^47^ Moreover, animal studies show that fenofibrate treatment increases hepatic TAG synthesis and hepatic steatosis suggesting an adverse effect of fibrates and PPARα activation in NAFLD.^48,49^

The reconstruction of the PPARα regulatory network permitted dynamic insights into the role of the PPARα regulome in both fatty acid metabolism and NAFLD. Simulation of lipid loading using just the GSMN (i.e. without PPARα-mediated regulation of gene expression) showed no increase in flux towards TAG synthesis. In contrast, addition of PPARα signalling to the GSMN predicts an increase in TAG synthesis. Such data is consistent with previous studies identifying PPARα as a key regulatory node in lipid metabolism and in diseases manifesting steatosis.^50,51^ However, the specific role of PPARα in the pathogenesis and progression of steatosis is confounded by conflicting reports in the literature. The finding that PPARα activation appears to have a detrimental role in hepatic steatosis may go some way to explaining the lack of success in trying to exploit PPARα as a therapeutic target in NAFLD treatment.^49,52,53^ However, if one considers that the function of the complement of genes regulated by PPARα is largely associated with fatty acid metabolism, de novo lipogenesis and lipid transport it becomes clear that a PPARα naive system would have a reduced capacity for TAG accumulation. Activation of PPARα results in the induction of expression of multiple genes with direct and indirect roles in TAG synthesis. As such, the regulatory competent model adapts to the increased concentrations of fatty acids in such a way that it can store this excess as relatively safe TAG within lipid droplets.^54^

The in vitro experiments, designed to test the hypothesis generated by the in silico experiments with the PPARα regulome model, are in agreement with simulated results. In the presence of the PPARα antagonist GW6471, a reduced accumulation of lipid droplets in the early stage of the steatotic response was observed in the cultured hepatocytes. We note that while the model simulations show a stark difference between the regulated and unregulated systems, the in vitro cell system exhibits a subtler, but statistically significant, difference. This is not unexpected given the qualitative nature of this model, driven by the scarcity of kinetic parameters to describe the induction and turnover of 91 PPARα target genes. As such, the in silico model accurately reproduces the behaviour of the network, but not the absolute quantitative changes. The endpoints that are being monitored in both the in vitro and in silico models are subtly different but allow us to compare the response of the two systems. Finally, while our in silico model is limited to PPARα-mediated regulation of gene expression, our in vitro system is regulated through the activity of an interlinking network of transcription factors. PPARα is not the only mediator of an adaptive response to excess fatty acid in hepatocytes.^52,55^ Future work to reconstruct other critical regulatory systems will allow us to explore the role of each of these transcription factors through simulations such as those shown here with PPARα. However, a significant advantage of the use of modelling and simulation, and the QSSPN approach, is the facility to not only study these regulatory factors in isolation, but also as integrated, cross-talking networks of transcription factors.

It is often considered that the emergence of NAFLD is the result, at least in part, of dysregulation of critical metabolic/transporter mechanisms. Dysregulation implies that the system is no longer being regulated correctly either through interference in agonist binding to the transcription factor or through suppression of transcription factor expression.^52,55^ However, two conclusions from our work suggest that this is not the case for PPARα signalling during the hepatocyte response to lipid stimulation. First, the in vitro data shows that although PPARα pathways are identified in our omics pathway analysis, this is due to activation of these pathways and not due to regulation of PPARα protein expression itself. Second, the in silico simulations integrating the PPARα regulatory network cause an increased flux towards TAG. Rather than the traditional interpretation of dysregulation, we suggest that, in the case of lipid loading, dysregulation of PPARα signalling is a result of persistent and repeated activation of the receptor. Given that the model predicts a continuing regulatory ‘ripple effect’ even after the clearance of the agonist, it is clear that regulation is not simply an ‘on’ ‘off’ response limited by interaction of agonist and receptor. It should be noted that the presented simulations are not in real time and so it is not possible to determine how long the reconstitutive phase persists. However, if this phase persists sufficiently that the basal state is not reached before the next signalling event, then repeated signalling would result in a form of dysregulation that could lead to disease.^56^ The current model only simulates regulation through a single transcription factor. In reality, nuclear receptors form a complex interactome that acts to coordinate the biological response to chemical challenge. As such, expansion of the current model to include multiple transcription factors may enhance the prediction robustness. Likewise, both in silico and in vitro models represent isolated hepatocytes; hence, their ability to reproduce a systemic disease that affects other tissues as well may be limited. Future work should replicate the interplay of multiple cell types that reside in the liver, such as hepatic stellate cells and Kupffer cells, as well as the interaction between different organs.

In summary, this work demonstrates that QSSPN permits both integration of qualitative and quantitative regulatory models with GSMNs. The multi-scale models herein, reproduce sugar and fat metabolism in silico, generating hypotheses that can be confirmed in vitro. Our data supports the absence of a differential lipogenic effect between glucose and fructose. In addition, we have provided mechanistic insight into the role of the PPARα regulome in the early metabolic reprogramming of hepatocytes following lipid loading.

## Methods

### Model reconstruction and QSSPN simulation

#### Multi-scale modelling of hepatic monosaccharide metabolism

This model integrated three components: a hepatocyte-specific GSMN,^19^ biologically realistic monosaccharide transport kinetics, and insulin signalling.^26^ To represent in vivo-like transportation of glucose and fructose, Michaelis–Menten kinetics were utilised to map substrate concentrations to fluxes within the activity list of the monosaccharide constraint places. All parameters for the kinetics were sourced from the literature with a greater weight given to values derived from whole-cell uptake experiments, as opposed to recombinant systems; further detailed in the Supplementary Methods. Monosaccharide transport rates were converted to mmol/g DW/h based on the assumptions outlined in Tables S1 and S2, and the activity list for glucose and fructose transport constraint places were set as symmetrical.

As further detailed in the supplementary methods, a Petri net formalism was used to represent the insulin signalling network^26^ using the Petri net editor software Snoopy 2^57^ (Fig. S1). The Petri net formalism was validated in the following manner: the original BioModels SBML file (MODEL1204060000) was implemented in COmplex PAthway SImulator (COPASI)^58^ and compared to QSSPN simulations, with consistent results across simulators (Fig. S2).

#### Genome-scale metabolic network

The transport and signalling network was integrated with a hepatocyte-specific GSMN, HepatoNet1,^19^ using QSSPN^24^ with the multi-formalism interaction network simulator (MUFINS) software.^25^ As described previously,^23^ specific adjustments were made to better detail metabolic pathways of interest accurately (Table S3). Within the modified GSMN, the total number of metabolites and reactions totalled 778 and 2542, respectively. A biomass function was used as a simulation constraint, representing the basic metabolic requirements of a human cell, including glucose and ATP (Table S5). Additional flux constraints were set to represent physiological relevant kinetic activity of the first steps of monosaccharide metabolism (Table S3). The external metabolite export set was also modified to represent the nutrient composition of cell culture medium (Table S4), and constrained by using maximal and minimal consumption/release values from NCI-60 cell lines,^27^ as done previously.^23^

#### Impact of the PPARα regulome on hepatocyte lipid loading

This model was reconstructed using the paradigm described above for the monosaccharide model, integrating a hepatocyte-specific GSMN and a de novo PPARα gene regulatory network. The putative complement of PPARα target genes was reconstructed using a Petri net formalism, based upon the published literature and online data repositories (KEGG, IUPHAR, PID). PPARα-regulated genes were represented using a rule-based model of gene expression, expanding on our previously described gene expression model (Figure S3).^24^ PPARα target genes were then systematically coupled to HepatoNet1, with the status of protein PN nodes modulating the flux bounds of corresponding metabolic network fluxes (Table S7). As described above, a biomass function was used as a simulation constraint, and the HepatoNet1 physiological import and export sets were modified to represent the nutrient composition of cell culture medium for better comparison with our in vitro model (Table S4).

The reconstructed QSSPN model was used to simulate a dynamic, PPARα competent regulatory model and a fixed gene expression model. The number of simulation steps was optimised so that the metabolic network returned to a basal, steady-state flux after treatment.

### In vitro experiments

#### Cell Culture

Human hepatocellular carcinoma-derived cell lines (Huh7, JCRB, Japan; HepG2, ATCC, UK) were maintained at 37 °C in a 5% CO_2_ air, humidified environment and regularly confirmed mycoplasma-free through a PCR assay. Cells were routinely seeded at 30,000 cells/cm^2^ and sub-cultured in Dulbecco’s modified Eagle’s medium (DMEM) supplemented with 10% foetal bovine serum, 1% non-essential amino acids, 2 mM l-glutamine, and 100 U/mL penicillin and 100 U/mL streptomycin (Lonza, UK) for 72 h prior to treatment, and passaged by trypsin treatment to represent independent experiments. After supplementation, final glucose concentrations of the media were estimated as 0.87 g/L (4.8 mM) and 3.92 g/L (21.7 mM) representing low and high glucose exposure, respectively. For the monosaccharide assays, glucose-free DMEM was supplemented with either 25 mM glucose or 25 mM fructose, with and without 100 nM insulin. In addition to the routine supplements listed above, these media also contained 1 mM sodium pyruvate (Gibco, Fisher Scientific, UK).

For pAKT measurements, cells were treated with vehicle (*n* = 3), 1, or 100 nM of insulin (*n* = 4) in serum-free DMEM for 15 min. For the fatty acid treatments, fatty acids were first solubilised in dimethyl sulfoxide (DMSO) and conjugated to 5.56% (v/v) fatty acid-free bovine serum albumin in a 1-h incubation at 37 °C with periodic vortex mixing prior to addition to serum-free DMEM. Final concentrations of DMSO in treatment media were maintained at 2% (v/v); a dose determined experimentally not to be cytotoxic by both LDH and MTT assays (in contrast, 2% DMSO is toxic to HuH7 cells, Fig S8). Cells were cultured with fatty acids for up to 24 h and sampled at required intervals. In the antagonist experiments, cells (*n* = 4) were concomitantly treated with 400 µM OA in the presence or absence of 10 µM PPARα antagonist GW6471 (Sigma Aldrich, UK) for 2 h.

#### Lipid and sugar assays

The fluorescent, lipophilic dye, Nile red (Sigma-Aldrich, UK) was used to detect intracellular lipid after OA and sugar treatments (both, *n* = 5). Briefly, an automated cell counter (T20; Biorad, UK) was used to count and collect 500,000 cells after trypsin treatment. Cells were centrifuged for 5 min at 500 × *g* at room temperature and the supernatant was removed. Cells were re-suspended in 500 μL of 1 μM Nile red in PBS pre-warmed to 37 °C and incubated for 10 min in darkness at 37 °C. Cells were centrifuged again and the supernatant removed before adding 500 μL of pre-warmed PBS. Three technical replicates of 100 μL of Nile red stained cells suspended in PBS per well were incubated for 2 min and shaken for 30 s at 37 °C before reading fluorescence (λex 485–12 nm, λem 520 nm) on a multi-mode plate reader (BMG LABTECH, Germany).

Sugar consumption was monitored by sampling the culture media at 0 h (*n* = 3) and at 24 and 48 h of 25 mM glucose (*n* = 5) and fructose (-insulin, *n* = 5; + insulin, *n* = 4) containing media, or after OA treatment (*n* = 3); and quantified by using glucose and fructose assay kits (Abcam, UK).

#### RNA and protein analyses

For qRT-PCR measurement of PPARα expression, cells (*n* = 3) were homogenised in Trizol (ThermoFisher, UK) and total RNA isolated as per the manufacturer’s instructions. Quantity and purity was assessed by absorbance spectroscopy (λ 260 nm, 280 nm and 230 nm); integrity was confirmed via gel electrophoresis. RNA samples were DNase treated prior to first strand synthesis (SuperScript III; ThermoFisher, UK) and SYBR green based PCR run using custom designed primers (MWG Eurofins, Germany; Supplementary Table S6) on an ABI7500 (ThermoFisher Scientific, UK).

For protein isolation, treated cells (OA, *n* = 3; insulin, *n* = 3–4) were homogenised in radio immunoprecipitation assay buffer (Sigma, UK) containing protease and phosphatase inhibitors (Pierce, UK). Cell homogenates in RIPA buffer were spun through a Qiashredder column (Qiagen, UK) to break up insoluble cellular debris. Protein was quantified by a BCA assay (Pierce, UK) and 20 µg from each sample subjected to SDS-PAGE and subsequent immunoblot analysis. Blots were probed for PPARα (1:250; 15540-1-AP Proteintech, UK), COXIV (1:5000; Ab16056 Abcam, UK), pAKT or AKT (pAKT #4060; AKT #2920; both: 1:2000; Cell Signalling Technology, The Netherlands). Immobilised proteins were quantified using secondary antibodies conjugated to near infra-red flurophores and visualised using the odyssey system (LI-COR, UK). All blots within each respective experiment were processed in parallel.

#### Data analysis

Data were represented as mean ± standard error of the mean (SEM), unless otherwise specified. One-way ANOVA with Tukey’s or Dunnett’s test post hoc; two-way ANOVA with either Tukey’s or Bonferroni’s test post hoc; and two tailed t-test with Welch’s correction were performed, as appropriate, to detect statistical significance between groups. The level of statistical significance was set at *P* < 0.05.

### Software and Code

All models were built using the Petri net edition software Snoopy (http://www-dssz.informatik.tu-cottbus.de/DSSZ/Software/Snoopy) and implemented by QSSPN (original implementation http://sysbio3.fhms.surrey.ac.uk/qsspn/index.html; now expanded within MUFINS^25^ package). All files that support the findings of this study are available from the corresponding author upon reasonable request.

### Data availability

The MS proteomics data have been deposited to the ProteomeXchange Consortium (http://proteomecentral.proteomexchange.org) via the PRIDE partner repository with the dataset identifier PXD001442.^31^ All other data that support the findings of this study are available from the corresponding author upon reasonable request.

## Electronic supplementary material

Supplemental Information

## Electronic supplementary material

**Supplementary information** accompanies the paper on the *npj Systems Biology and Applications* website (10.1038/s41540-018-0070-3).
