# Supplementary material for: Multi-scale, whole-system models of liver metabolic adaptation to fat and sugar in non-alcoholic fatty liver disease
Source: NPJ Syst Biol Appl. 2018 Aug 20;4:33. doi: 10.1038/s41540-018-0070-3 (PMC6102210; doi:10.1038/s41540-018-0070-3)
Supplement: Supplementary file 1 — Supplemental Information [file 41540_2018_70_MOESM1_ESM.pdf]

SUPPLEMENTARY INFORMATION

Multi-scale, whole-system models of liver metabolism adaptation to fat and sugar in non-alcoholic fatty liver disease.

Maldonado EM, Fisher CP, Mazzatti DJ, Barber AL, Tindall MJ, Plant NJ, Kierzek AM & Moore JB[1].

[1]Corresponding Author: J. Bernadette Moore j.b.moore@leeds.ac.uk

SUPPLEMENTARY METHODS 2

SUPPLEMENTARY FIGURES 9

Figure S1. Modelling strategy overview. 9

Figure S2. The multi-scale model of hepatic monosaccharide metabolism 10

Figure S3. Import and validation of the insulin kinetic model into QSSPN. 11

Figure S4. The QSSPN gene expression model 12

Figure S5. Adjacency matrix heatmap 13

Figure S6. An unregulated exchange set heatmap. 14

Figure S7. 24 hour *in vitro* PPARα inhibitor experiments. 15

Figure S8 Cell viability of fatty acid treatment controls. 16

SUPPLEMENTARY TABLES 17

Table S1. Hepatocyte key assumptions. 17

Table S2. HepG2 macromolecular composition. 18

Table S3. HepatoNet1 modifications. 19

Table S4. External metabolite exchange set 20

Table S5. Generic biomass function used for simulations 22

Table S6. Custom primer sequences 24

Table S7 Target genes and mediated hepatic reactions 25

REFERENCES 51

SUPPLEMENTARY METHODS

Multi-Scale Model of Hepatic Monosaccharide Metabolism Reconstruction and QSSPN Simulation

The multi-scale model of the regulation of sugar uptake and its effect on global hepatocyte metabolism is composed of a fully parameterised ordinary differential equation (ODE) model of insulin signalling[1] and a hepatic genome scale metabolic network (GSMN)[2] constrained by consumption and release flux bounds derived from a metabolomics *in vitro* study.[3]

A Petri net formalism was used to represent the signalling network composed of monosaccharide transport and insulin signalling (**Fig. S2**). Reconstruction was performed with the Petri net editor software Snoopy 2 and run on Mac OS X; v1.13, build Apr 1, 2014.[4] Utilising the extended Petri net class, compounds were represented by places (circles), while transitions (squares) represented interactions in the model. Quasi steady-state Petri nets (QSSPN)[5] were used to connect molecular interactions that occur over different time scales. Fast metabolic interconversions represented as quasi steady-state fluxes (QSSF; HepatoNet1, **Fig. S2A**); and slower gene and signalling regulatory networks represented as dynamic transitions (DT; Petri net network, **Fig. S2B-F**). Constraint places in the DT set the flux bounds in the metabolic network through an activity list linking the number of tokens in the place to upper and lower bounds for specified reactions within the GSMN. Constraint places were also used to link qualitative and quantitative aspects of the DT (e.g. G6Pase). Transitions can be used to define the reaction rate constant for a reaction through the 'RATE' comment, with a rate of 1 being used as default if this is not present. In addition, the 'FLUX' comment can be used to extract real flux values from the simulated solution;[6] these transitions are indicated as red rectangles within **Figure S2**. Standard (point-ended arrows) and read (circle-ended arrows) edges were used to simulate consumption or catalyzation of the interactions, respectively. Edges were tagged to specify the activity of the pre-place, e.g. 'ACTIVITY 0' followed by 'END' denotes mass action activity. To represent *in vivo*-like transportation of glucose and fructose, Michaelis–Menten kinetics (**Eq. 1**) were utilised to calculate the fluxes within the activity list of the monosaccharide constraint places, such that

$$v = \frac{Vmax[S]}{Km+[S]}, \qquad \textbf{Eq. 1}$$

where v is the rate of the reaction, $Vmax$ is the maximum rate possible for the reaction, $[S]$ is the substrate concentration and K_M is the Michaelis–Menten constant which is unique to each substrate.

The $Vmax$ was set at 5 mmol/g DW/h for both glucose and fructose based on favouring whole cell uptake, rather than recombinant systems.[7,8,9,10] HepG2 cells are known to express more than one glucose transporter, e.g. GLUT1, GLUT2, GLUT3 and GLUT9,[11,12,13,14] but not

the main fructose transporter, GLUT5.[12,15] Glucose transport is symmetrical.[9] Therefore, the activity list for glucose and fructose transport constraint places are symmetrical in regards to the maximal import and export rates represented as lower and upper bounds, respectively. Monosaccharide transport rates[7,8,10] were converted to mmol/g DW/h based on assumptions outlined in **Table S1** and **Table S2**. The Michaelis–Menten constants were set at 17 mM for glucose and 66 mM for fructose based on GLUT2 transport properties as it is the predominate glucose and fructose transporter in hepatocytes.[11,16,17] Extrapolating from a Michaelis–Menten curve based upon these kinetic values, 30 activity step thresholds were generated. This ensures a high resolution over the substrate concentration range of 0 - 25 mM for each monosaccharide, which corresponds to the concentrations used during *in vitro* experimentation.

This unregulated model was expanded to include a regulatory signalling pathway through the integration of a model of insulin signalling[1] downloaded from BioModels (ID: MODEL1204060000, SBML L2 V4).[18] The kinetic insulin model was first implemented in COmplex PAthway SImulator (COPASI)[19] and simulated on a Mac OS X; v4.15, Build 95 to ensure the model could successfully replicate the behaviours reported in the original paper. Briefly, simulations were carried out as a deterministic (LSODA) time course over a duration of 480 minutes. COPASI time course simulations (**Fig. S3B**) resulted in similar behaviour as seen in the original publication (**Fig. S3A**). The insulin model was converted into a Petri net formalism using Snoopy (**Fig. S2F**). Large places denoted signalling molecules, medium sized places represent reaction rates and the smallest places represent degradation. Places that are shaded grey represent clones of individual places to enhance visualisation: all clones for a single entity contain the same concentration/flux. Transitions were labelled as the reaction rate IDs in the original publication, and were parametrised by their pre-place. The insulin model simulated in QSSPN was consistent with the model simulated in COPASI, producing identical results (**Fig. S3B, C**).

The kinetic insulin model was then coupled with the HepatoNet1 GSMN. Within the original kinetic insulin model, only one gene product had a direct link to a metabolic reaction in HepatoNet1: G6Pase (r0396, H2O(r) + Glucose-6P(r) → Glucose(r) + Pi(r)). To enhance the regulatory connection between the insulin regulatory network and the metabolic network, phosphorylated glycogen synthase kinase (pGSK)3β was designated as the active glycogen synthase (GYS; r1388, 3 UDP-glucose(c) + Glycogenin-G8(c) → Glycogenin-G11(c) + 3 UDP(c)).[20,21] Comments in the original publication also noted that another compound, phosphoenolpyruvate carboxykinase (PEPCK), had a similar behaviour to G6Pase but it was not included in the original kinetic model.[21,22] Therefore, the activity of G6Pase was used to represent the activity of PEPCK and connected with HepatoNet1 reactions r0123-4 for both

cytosolic (c) and mitochondrial (m) compartments of reaction; GTP + OAA → GDP + PEP + CO2.

The HepatoNet1 GSMN,[2] representing human liver whole-cell metabolism, was downloaded from the BioModels database (MODEL1009150000, SBML L2 V4).[23] All fluxes were checked to ensure the direction of reactions in the stoichiometric matrix. All reversible fluxes were adjusted to a minimum flux of -1 and a maximum flux of 1; irreversible reaction fluxes were adjusted to 0 to 1 or -1 to 0 depending on directionality.

Specific adjustments were made to HepatoNet1 to better detail metabolic pathways of interest accurately. The polyol pathway was included as this has been found to be a potential influence on the development of hepatic steatosis[24] and is part of fructose metabolism in the liver.[25] Namely, HepatoNet1 reaction IDs: r0206 (ketohexokinase, KHK, EC 2.7.1.3), r0252 (triokinase, TRIOK, EC 2.7.1.28), and r0554 (adolase B, ALDOB, EC 4.1.2.13) were adjusted as shown in **Table S3**. In addition, a metabolite, sorbitol, sorbitol dehydrogenase (SORD) and aldo-keto reductase family 1, member B1 (AKR1B1) reactions were added to represent the polyol pathway. The hepatic protein (protein score) and RNA (fragments per kilobase of exon per million fragments mapped, FPKM) expression of SORD and AKR1B1 in human liver were confirmed by expression data from Human Protein Atlas[26] and HepG2 cells. A reaction was also added to represent the hepatic fructose sodium co-transport reaction.[27] Thus, the total number of metabolites is 778 and the total number of reactions is 2542 within the modified GSMN.

A biomass function was used as a simulation constraint to ensure the presence of the essential building blocks (amino acids, nucleoside triphosphates and deoxynucleotide triphosphates) for biomass were available; representing the basic metabolic requirements of a human cell. This function was adapted based on a published whole human metabolism GSMN, Recon 2, biomass[28] (**Table S5**). FBA simulations before and after the modifications listed above, and using the same external metabolite constraints **(Table S3),** were performed: BIOMASS optimal maximisation flux was not altered by the modifications: FBA = 0.03152. Biomass doubling time estimations for HepG2 cells range from approximately 20 to 60 hours.[29,30,31,32] To reflect this, the BIOMASS lower and upper flux bounds were set at 0.01666667 and 1000.0, respectively, representing a minimal doubling time of 60 hours.

Additional constraints were included to represent the physiologically relevant kinetic activity of the first steps of glucose and fructose metabolism. Flux constraints (mmol/g DW/hour) were set as a ratio of fluxes. As a conservative measure, flux ratio assumptions were based on a hexokinase Michaelis-Menten constant K_M for glucose and fructose phosphorylation at carbon-6, as hexokinase II is the most likely expressed hexokinase in HepG2 cells.[14] The hexokinase K_M for glucose and fructose phosphorylation were estimated

as 0.047 mM and 11.5 mM, respectively.[33] Thus, hexokinase II flux ratio is set for the phosphorylation of glucose specific reactions (r0353-5) at 1, and reactions specific to fructose phosphorylation (r0356-8) was set at 0.005. The polyol pathway flux was also adjusted. The K_M for glucose phosphorylation by hexokinase was estimated to be 0.047 mM, while the K_M of the first reaction in the polyol pathway by aldose reductase enzyme was estimated as 65.8 mM.[34,35,36] Thus, hexokinase phosphorylation of glucose was set as 1 (as above) and the flux of the reaction specific to AKR1 was set as 7.14 x 10^{-4}. For computational purposes, the TAG production reaction (r1223) was adjusted as a positive flux as QSSPN simulations can only maximise positive objective function flux values. Additionally, TAG production reaction (r1223) lower and upper bound values were set as 0 and 10, respectively, so as not to mask the optimisation flux if above 1.

To represent the nutrient composition of the culture medium, transport reactions were included as an external exchange set. The HepatoNet1 physiological import and physiological export set (PIPES) was modified to represent the nutrient composition of cell culture medium as shown in **Table S4**. The fluxes of the external metabolite set were constrained by setting the lower and upper bound fluxes as the maximum consumption and release rates derived from the NCI-60 cell lines consumption and release of metabolites dataset (CORE)[3], respectively, as previously done in [37].

Model Simulation

The QSSPN algorithm integrates QSSF set by the metabolic network with DT represented by the Petri net network.[5,6] QSSF and DT interaction sets are connected by two classes of Petri net places: constraint places set flux bounds in QSSF, translating place status into flux bounds; objective places represent metabolic outputs of the QSSF network. Specialised transitions are introduced as FLUX transitions so to extract flux values from simulation solutions. The QSSF is the metabolic network, i.e. HepatoNet1, represented as a stoichiometric matrix of which during simulation, the network is constrained by mass balance and ultimately is at steady-state at each time step during the simulation. While the quantitative bounds substantially limit solution space,[37] alternative flux distributions within the whole-cell metabolic model are possible. To evaluate a full range of quantitative model behaviours, we formulated a dynamic Flux Variability Analysis (dFVA) protocol (shown below), applying for the first time the well-established Flux Variability Analysis[38] in a quasi-steady state dynamic simulation. A dFVA simulation produces two time courses corresponding to the maximal and minimal value of a reaction flux that can sustain the maximal value of the metabolic capability (objective function) under investigation. This provides insight into model behaviours that are feasible (and not feasible) given the knowledge represented in the ODE model, stoichiometric

model (GSMN) and the consumption/release constraints. Comparison of the dFVA results with experimental data thus evaluates the extent to which current molecular mechanistic knowledge recapitulates observed system dynamics. In summary, the model presents a novel method, dFVA.

The dFVA approach employs the following steps:

i. Calculate the constraint and the objective nodes;
ii. Update the metabolic model accordingly to (1);
iii. Optimise the metabolic model using *maximum* optimisation dFBA;
iv. Update the Petri net objective node according to the new objective;
v. Repeat (1-4) until simulation time has ended; and
vi. Repeat (1-5), except using *minimum* optimisation dFBA for step 3.

The majority of dFVA simulations were run with a maximal time step of 0.01. However, this proved to be computationally expensive when simulating the integrated model of insulin and HepatoNet1 optimising for TAG production (as the objective function). Therefore, this setting was adjusted to 0.1. Under these conditions, simulations were still of sufficiently high resolution to allow for examination of the network behaviour.

Sugar Consumption Assay

Sugar consumption was monitored by sampling culture media over time and quantified by using glucose and fructose assay kits (Abcam, UK). Both glucose and fructose were stable in medium over the experimental period, and no extracellular degradation was expected. The disappearance of monosaccharide from the medium is highly suggestive and was regarded as cellular uptake and consumption. Briefly, medium or diluted medium (medium:assay buffer, 1:4, *v*/*v*) was diluted with assay buffer (1:24, *v*/*v*). The prepared medium was then incubated with glucose reaction mix (assay buffer: probe: enzyme mix, 23:1:1, *v*/*v*/*v*) or fructose reaction mix (assay buffer: probe: enzyme mix: fructose converting enzyme, 18:1:1:5, *v*/*v*/*v*/*v*) at a 1:1 (*v*/*v*) ratio mixture for 30 minutes or 1 hour, respectively, in darkness at 37°C. Absorbance for both assays were read at 570nm by a multi-mode plate reader (BMG LABTECH, Germany).

Insulin Sensitivity by pAKT

Cells were lysed with radioimmunoprecipitation assay buffer containing ×1 Halt™ protease and phosphatase inhibitor cocktail. Protein concentrations were determined by bicinchoninic acid (BCA) assay (Fisher Scientific, UK). As a positive and negative control for pAKT detection, AKT control cell extracts (New England Biolabs Ltd, UK) were included. Proteins

were separated by molecular weight via sodium dodecyl sulphate - polyacrylamide gel electrophoresis (SDS-PAGE). Gels containing 6% acrylamide/bis [37.5:1] for stacking and 12% for resolving were loaded with 20 µg of protein per lane, and resolved for 1 hour at 180 V. Proteins were wet-transferred onto PVDF membranes for 2 hours at 300 mA. Membranes were blocked with 0.1% BSA in tris-buffered saline (TBS) for 1 hour at room temperature. Blots were then probed with primary rabbit anti-pAKT (Ser473, D9E) and mouse anti-AKT (pan, 40D4), both at 1:2000 (New England Biolabs, UK) in TBS-0.2% Tween® 20 (T) overnight at 4°C. After washing for 5 minutes thrice (washing buffer: TBS-0.1% T), blots were probed with donkey anti-mouse IRDye 680RD and donkey anti-rabbit IRDye 800CW at 1:10000 (LI-COR Biosciences, UK) in TBS-0.2% T-0.01% SDS for 1 hour at room temperature in darkness. Membranes were washed again with washing buffer for 5 min thrice in darkness, rinsed twice with TBS and air-dried in darkness prior to blot visualisation on LI-COR ODYSSEY CLx (LI-COR Biosciences, UK) and quantified by Image Studio v3.1, software v1.0.11.

PPARα Regulome Reconstruction and QSSPN Simulation

Pathway Enrichment Analysis

Enriched pathways (P<0.05), within the transcriptomics and proteomics datasets described below, were identified against the KEGG[39] and BIOCARTA[40] databases using the DAVID[41] online bioinformatics suite. Results from these analyses were systematically combined and quantitatively represented using a hive plot formalism.[42]

Transcriptomics

Microarray experiments were performed as previously described.[43] Briefly, Huh7 cells were incubated with 100 µM or 200 µM of palmitic acid in serum-free DMEM for 24 hours. Cells were homogenised in Trizol (ThermoFisher, UK) and total RNA isolated as per the manufacturer's instructions. Total RNA quantity and purity was assessed by absorbance spectroscopy (λ 260nm, 280nm and 230nm); integrity was confirmed using the Agilent 2100 bioanalyser and the RNA 600 LabChip kit prior to hybridisation to human gene expression microarrays (Agilent Technologies, UK). Differential expression of genes (≥1.5 fold relative to controls, P<0.05) were identified in both treatment groups and taken forward for pathway enrichment analysis.

Proteomics

Details of the *in vivo* proteomics study have been described in detail elsewhere.[44] Briefly, C57BL6 and APOE-/- mice were fed either high-fat or normal chow diets for 12 weeks after

which animals were sacrificed, livers were resected, homogenised and protein isolated and enriched for cytoplasmic and membrane proteins. Fractionated samples were iTRAQ labelled for proteomics analysis by mass spectrometry. Proteins identified as being differentially expressed (relative to wild-type animals fed a normal diet) were carried forward for pathway enrichment analysis.

PPARα Model Simulation

Simulation of the PPARa model was performed as previously published[5] with the exception for updates to the gene expression model (**Fig. S4**), the biomass function (**Table S5**), and exchange set (**Table S4**) detailed herein.

SUPPLEMENTARY FIGURES

FIGURE S1

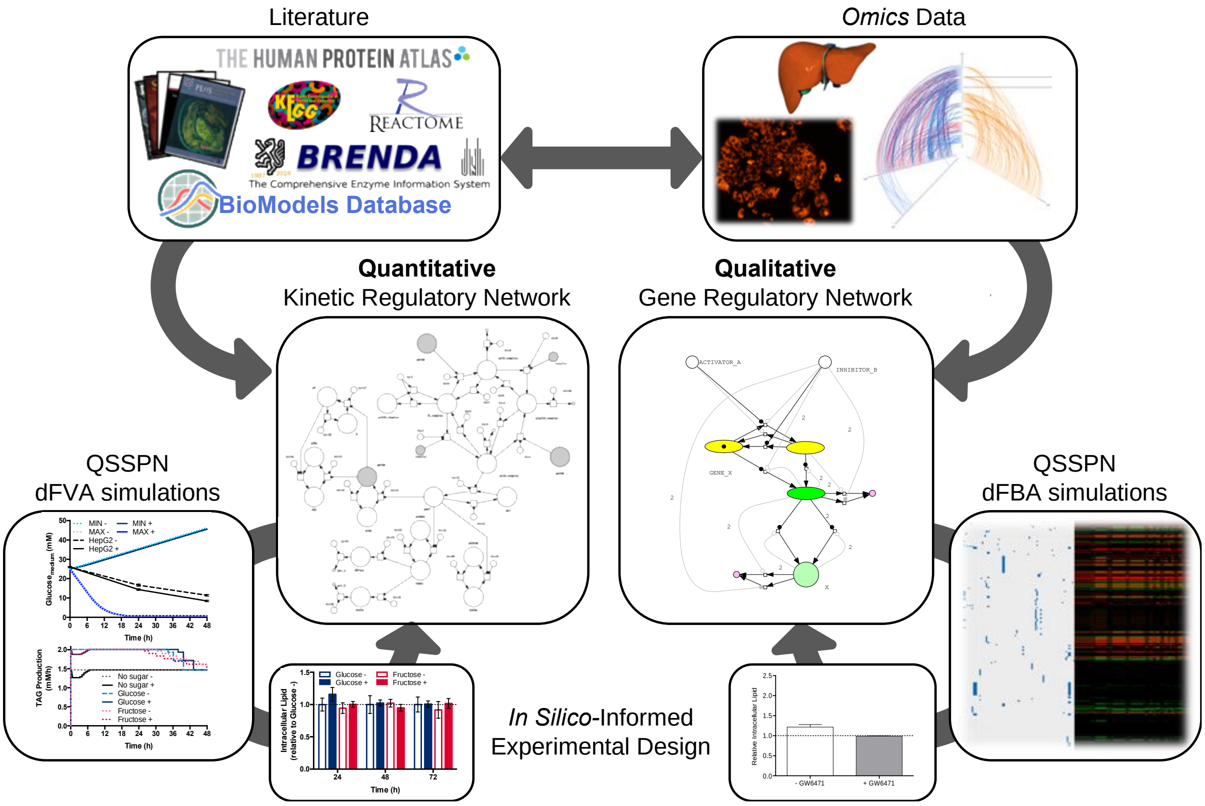

Figure S1. Modelling strategy overview. The computational model construction incorporates information from both in-house experimental data and literature. Although not exclusively, literature-based data was incorporated into our quantitative kinetic regulatory network, while in-house omics data was also used to aid the qualitative gene regulatory network construction. The quantitative and qualitative model is simulated to deliver outputs that can be used to compare to and/or inform experimental results. This is an iterative modelling approach as experimental results can then be used to update model design to deliver predictions more representative of known biology.

FIGURE S2

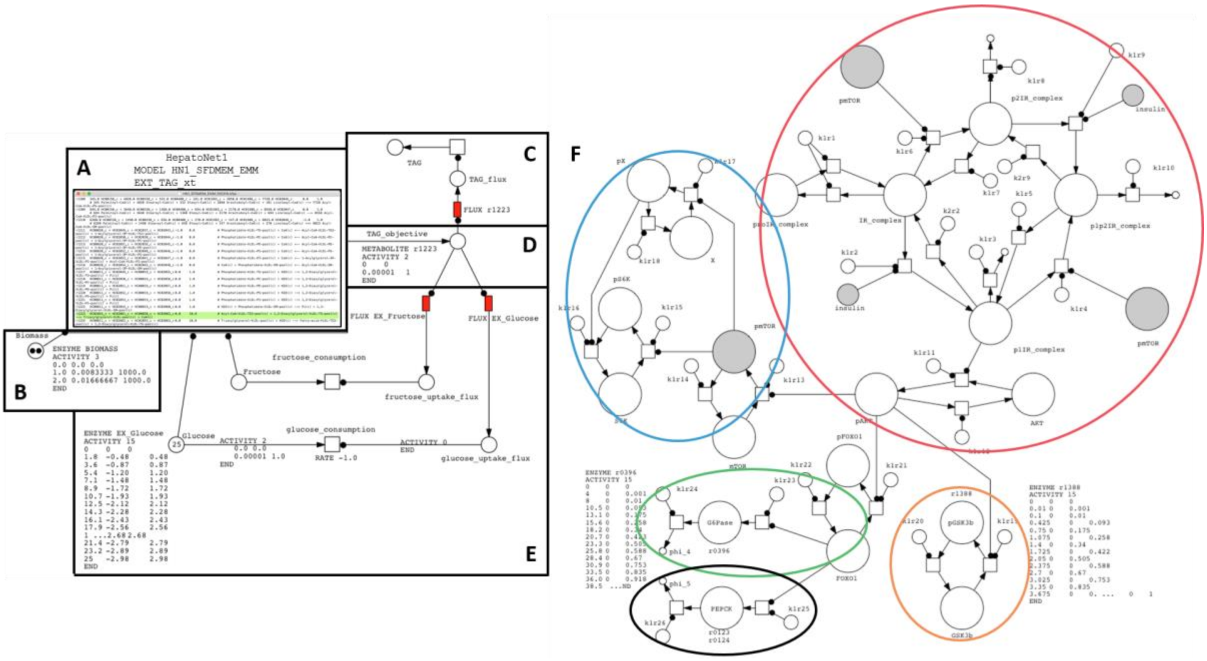

Figure S2. The multi-scale model of hepatic monosaccharide metabolism as viewed in the Snoopy graphical user interface. **A** Liver specific GSMN, HepatoNet1 with the modifications as outlined in Table S3. **B** A biomass constraint to represent a 'healthy hepatocyte' (Table S4). **C** Monitoring outputs, e.g. TAG production. **D** The objective function. **E** The sugar transport system. **F** The insulin kinetic model reconstructed in a Petri net formalism with coloured ovals used to highlight modules used by Kubota and colleagues (2012)[1]; the PEPCK module in black oval was interpreted as described in supplementary methods.

FIGURE S3

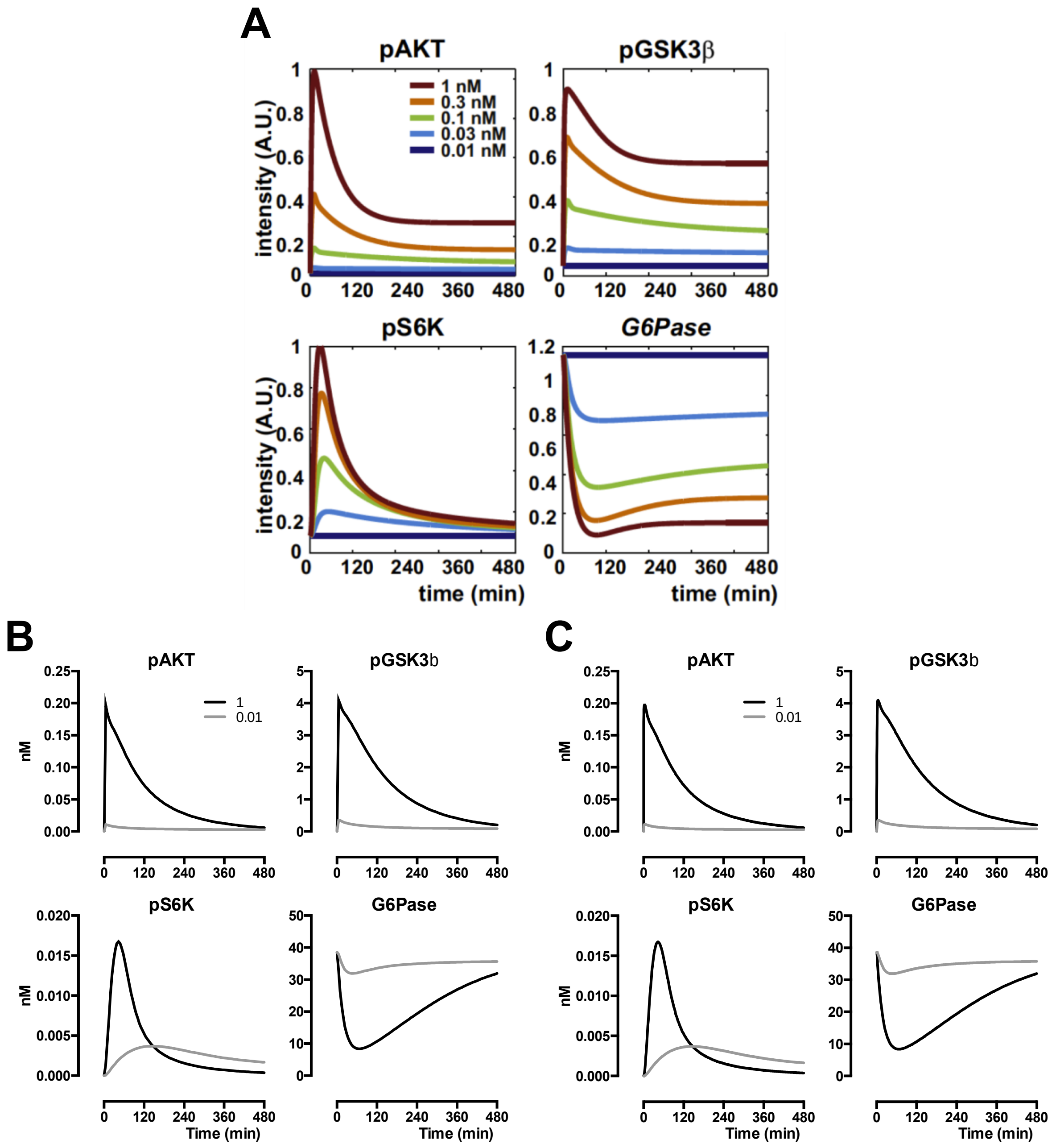

Figure S3. Import and validation of the insulin kinetic model into QSSPN. **A** Simulation results as presented from the original publication[1], and the results of the same kinetic model ran in **B** COPASI and **C** QSSPN with no notable differences between the simulations.

FIGURE S4

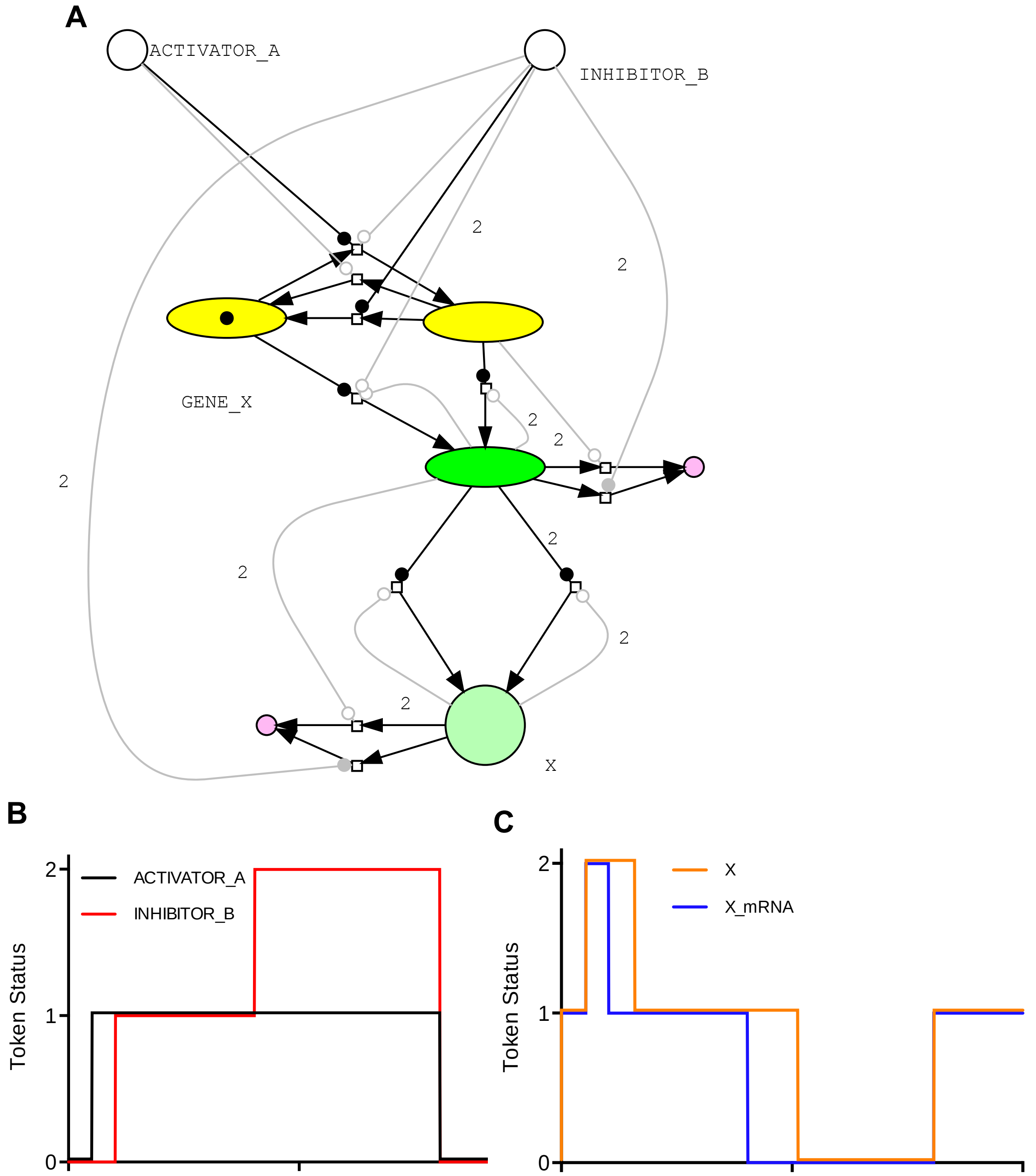

Figure S4. The QSSPN gene expression model simulating regulation of the expression of a hypothetical gene 'X'. **A** The gene expression model represented as an extended Petri net formalism incorporating systems biology graphical notation; **B** Token status of 'Activator_A' and 'Inhibitor_B' place nodes over simulated trajectory; **C** Expression of a hypothetical gene 'X' at the transcript and protein level over a simulated trajectory in response to activator and inhibitor status.

FIGURE S5

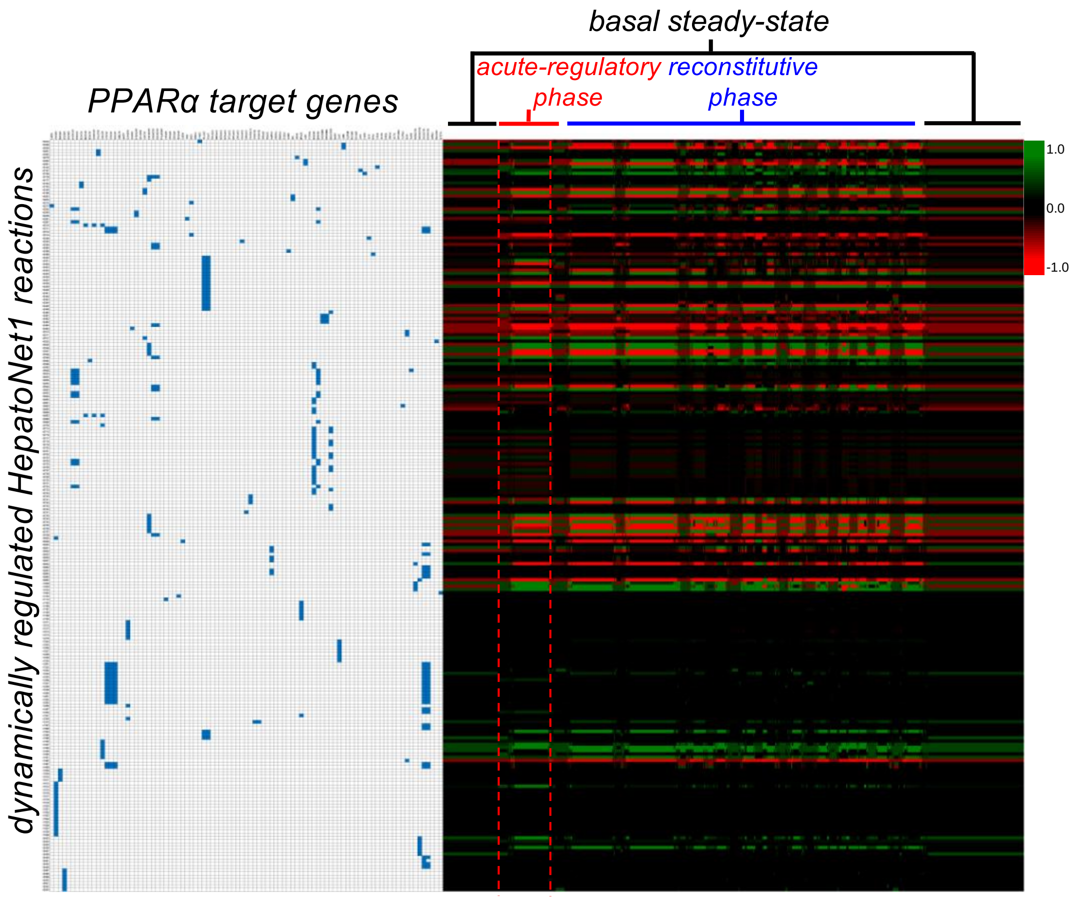

Figure S5. Adjacency matrix heatmap showing the reconstructed PPARα regulome connected to HepatoNet1 metabolic network fluxes and a single, representative simulated trajectory. The left panel indicates the mapping between the 91 PPARα target genes (alphabetical from left to right and per **Table S7**) and the 233 metabolic/transport reactions within the GSMN their encoded proteins catalyse. The right panel indicates flux through each reaction over the course of a simulation. Positive flux values are shown in green, negative flux values in red with simulated time progressing left to right; the simulated fatty acid treatment is indicated by the dashed redlines (TX window).

FIGURE S6

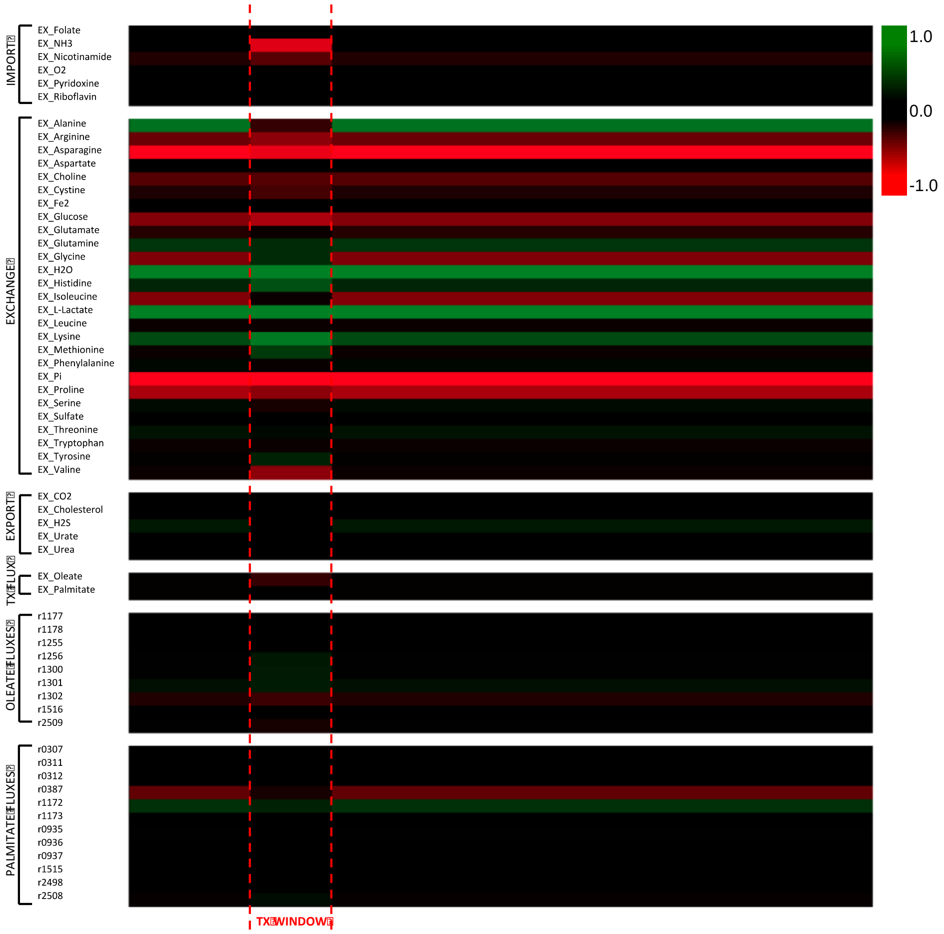

Figure S6. An unregulated exchange set heatmap. A single; single simulated trajectory heatmap of exchange set fluxes, treatment fluxes and all fluxes where palmitate and oleate are primary metabolites or products. Positive flux values are shown in green, negative flux values in red with simulated time progressing left to right; simulated fatty acid treatment is indicated by the dashed redlines (TX window). For these simulations, the PPARα regulome was disabled by setting the token status of the PPARα gene node to 0. As a result, PPARα is not expressed within the model and all target genes are permanently fixed at basal expression levels.

FIGURE S7

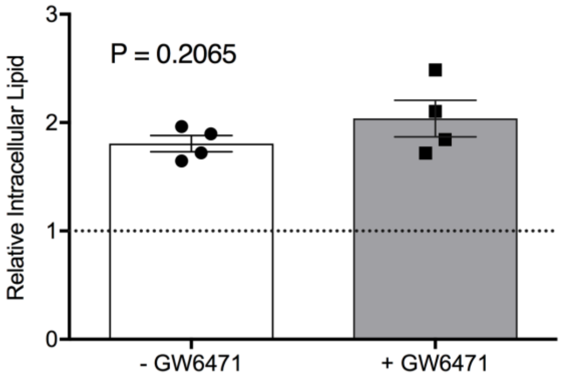

Figure S7. 24 hour *in vitro* PPARα inhibitor experiments. Relative intracellular lipid levels as quantified by Nile red fluorescence in HepG2 cells treated with 400 μM oleic acid for 24 hours ± PPARα antagonist GW6471 mean ± SEM (n=4) relative to vehicle and analysed using a two-tailed t-test with Welch’s correction.

FIGURE S8

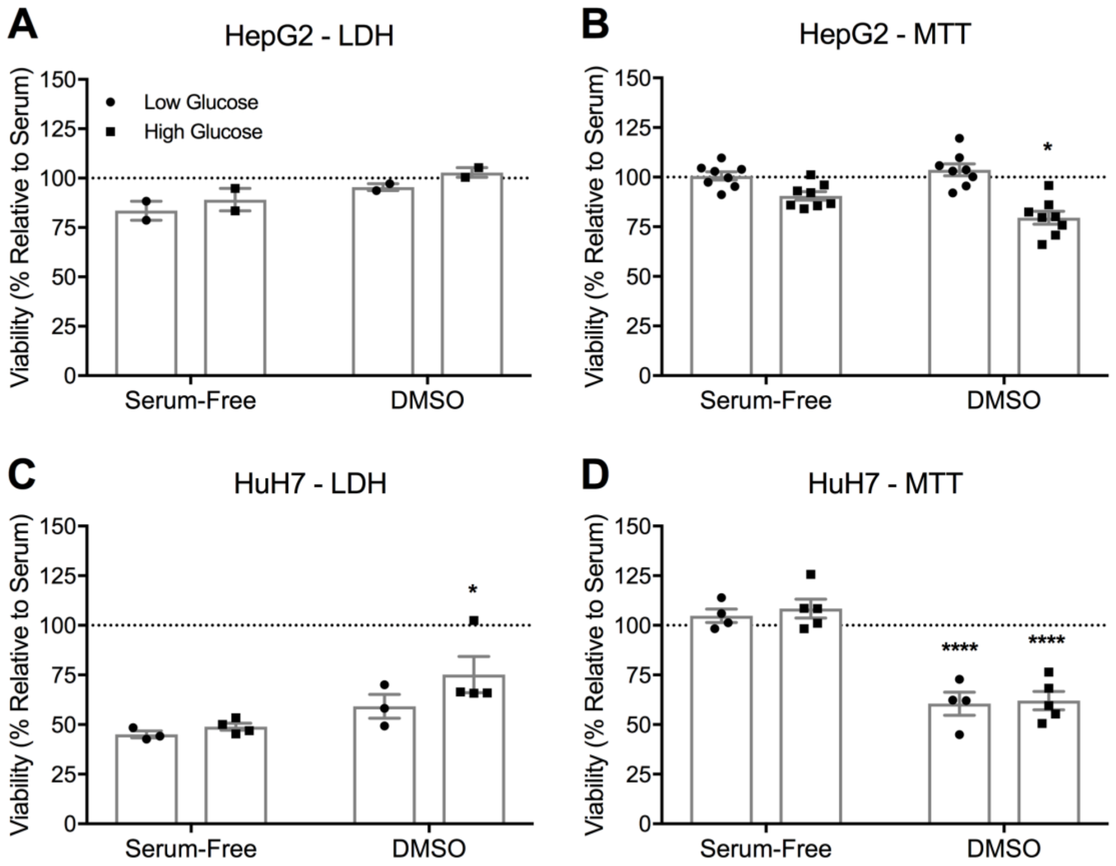

Figure S8 Cell viability of fatty acid treatment controls. Cell viability was detected by LDH (**A** and **C**) and MTT (**B** and **D**) assays in both HepG2 (**A** and **B**) and HuH7 cells (**C** and **D**) cultured in low (grey) or high (black) glucose DMEM after 24 h in serum-free or added 2 % DMSO. HepG2 viability by LDH (n=2) and MTT (n=8); HuH7 by LDH in low (n=3) and high (n=4) glucose; HuH7 by MTT in low (n=4) and high (n=5) glucose. Data are relative to serum cultured cells set at 100 % (dotted line) and shown as mean ± SEM. Two-way ANOVA with Bonferroni's test post hoc was used to detect differences between serum-free and added 2% DMSO treated cells with statistical significance indicated by * P < 0.05 and **** P < 0.0001.

SUPPLEMENTARY TABLES

TABLE S1

Table S1. Hepatocyte key assumptions.

Description	Estimations
Dimension	15 μm^3
Volume	3.375×10^{-9} mL
Density	1.03 g/mL
Total cell weight	3.476 ng
Dry weight *(1/3 total weight)*	1.1588ng
Wet weight *(2/3 total weight)*	2.25 pL

Estimations based on[45] and Table S2.

TABLE S2

Table S2. HepG2 macromolecular composition.

Macromolecules	% of dry weight
DNA	3.9
RNA	2.4
Carbohydrates	3.4
Lipids	18.0
Proteins	61.4
Rest	10.9

Composition was sourced from.[46]

TABLE S3

Table S3. HepatoNet1 modifications.

**Modified reactions**				
ID	HepatoNet1 Reaction	Modified Reaction		
r0206	ATP(c) + Fructose(c) ← ADP(c) + Fructose-1P(c)	ATP(c) + Fructose(c) → ADP(c) + Fructose-1P(c)		
r0252	ATP(c) + Glyceraldehyde(c) ↔ ADP(c) + GAP(c)	ATP(c) + Glyceraldehyde(c) → ADP(c) + GAP(c)		
r0554	Fructose-1P(c) → DHAP(c) + Glyceraldehyde(c)	Fructose-1P(c) ↔ DHAP(c) + Glyceraldehyde(c)		
**Added reactions**				
ID	Reaction			
r9000	Glucose(c) + NADPH(c) → NADP(c) + Sorbitol(c)			
r9001	NAD(c) + Sorbitol(c) → Fructose(c) + NADH(c)			
r9002	Fructose(s) + Na^+(s) → Fructose (c) + NA^+(c)			
**Constrained fluxes**				
ID	HepatoNet1 Reaction		Lower Bound	Upper Bound
r0356	Fructose(c) + ATP(c) → Fructose-6P(c) + ADP(c)		0.0	0.005
r0357	Fructose(c) + ATP(c) → Fructose-6P(c) + IDP(c)		0.0	0.005
r0358	Fructose(c) + ATP(c) → Fructose-6P(c) + dADP(c)		0.0	0.005
r9000	Glucose(c) + NADPH(c) → NADP(c) + Sorbitol(c)		0.0	0.000714
r1223	Acyl-CoA-VLDL-TG3-pool(c) + 1,2-Diacylglycerol-VLDL-TG-pool(c) → Triacylglycerol-VLDL-pool(c) + CoA(c)		0.0	10.0
r1224	Triacylglycerol-VLDL-pool(c) + H2O(c) → Fatty-acid-VLDL-TG3-pool(c) + 1,2-Diacylglycerol-VLDL-TG-pool(c)		0.0	10.0

Abbreviations: cytosol (c), sinusoidal space (s), glyceraldehyde 3-phosphate (GAP), dihydroxyacetone phosphate (DHAP), triacylglycerol (TG). The HepatoNet1 modifications were also used in Maldonado *et al.* (2017).[37]

TABLE S4

Table S4. External metabolite exchange set.*

Reaction ID	Reaction	Lower Bound	Upper Bound	Description
EX_H2O	HC00011_s = HC00011_s_xt	-1.0	1.0	H2O exchange
EX_O2	HC00017_s = HC00017_s_xt	-1.0	0.0	O2 import
EX_Pi	HC00019_s = HC00019_s_xt	-1.0	1.0	Pi exchange
EX_CO2	HC00021_s = HC00021_s_xt	0.0	1.0	CO2 export
EX_NH3	HC00024_s = HC00024_s_xt	-1.0	0.0	NH3 import
EX_Alanine	HC00048_s = HC00048_s_xt	-0.005500	0.130813	Alanine exchange
EX_Arginine	HC00065_s = HC00065_s_xt	-0.070285	0.005786	Arginine exchange
EX_Asparagine	HC00148_s = HC00148_s_xt	-0.037002	0.005679	Asparagine exchange
EX_Aspartate	HC00055_s = HC00055_s_xt	-0.017289	0.016442	Aspartate exchange
EX_Cystine[1]	HC00389_s = HC00389_s_xt	-0.608543	0.193094	Cystine exchange
EX_Glutamate	HC00034_s = HC00034_s_xt	-0.007645	0.193094	Glutamate exchange
EX_Glutamine	HC00067_s = HC00067_s_xt	-0.608543	-0.027744	Glutamine import
EX_Glycine	HC00045_s = HC00045_s_xt	-0.011301	0.023408	Glycine exchange
EX_Histidine[1]	HC00133_s = HC00133_s_xt	-0.608543	0.193094	Histidine exchange
EX_Isoleucine	HC00334_s = HC00334_s_xt	-0.043197	-0.002126	Isoleucine import
EX_Leucine	HC00121_s = HC00121_s_xt	-0.052230	-0.003955	Leucine import
EX_Lysine	HC00053_s = HC00053_s_xt	-0.038469	-0.005086	Lysine import
EX_Methionine	HC00075_s = HC00075_s_xt	-0.014031	-0.001399	Methionine import
EX_Phenylalanine	HC00081_s = HC00081_s_xt	-0.014610	-0.001669	Phenylalanine import
EX_Proline	HC00145_s = HC00145_s_xt	-0.002389	0.011564	Proline exchange
EX_Serine	HC00068_s = HC00068_s_xt	-0.067881	-0.004097	Serine import
EX_Threonine	HC00179_s = HC00179_s_xt	-0.024476	-0.002757	Threonine import

EX_Tryptophan	HC00080_s = HC00080_s_xt	-0.006982	-0.000055	Tryptophan import
EX_Tyrosine	HC00085_s = HC00085_s_xt	-0.021401	-0.002104	Tyrosine import
EX_Valine	HC00174_s = HC00174_s_xt	-0.031520	-0.003558	Valine import
EX_Choline	HC00112_s = HC00112_s_xt	-0.004596	0.000521	Choline exchange
EX_Folate	HC00396_s = HC00396_s_xt	-0.000046	0.000198	Folate exchange
EX_Nicotinamide	HC00149_s = HC00149_s_xt	-0.001838	0.000057	Nicotinamide exchange
EX_Pantothenate	HC00568_s = HC00568_s_xt	-0.000075	0.000005	Pantothenate exchange
EX_Thiamin	HC00316_s = HC00316_s_xt	-0.000662	0.000497	Thiamin exchange
EX_Urate	HC00310_s = HC00310_s_xt	-0.000306	0.000212	Urate export
EX_Glucose	HC00040_s = HC00040_s_xt	-1.0	1.0	Glucose exchange
EX_Fructose	HC00097_s = HC00097_s_xt	-1.0	1.0	Fructose exchange
EX_L-Lactate	HC00177_s = HC00177_s_xt	-1.0	1.0	L-Lactate exchange
EX_Fe2+	HC01846_s = HC01846_s_xt	-1.0	1.0	Fe2+ exchange
EX_Inositol	HC00135_s = HC00135_s_xt	-1.0	1.0	Inositol exchange
EX_Pyridoxine	HC00268_s = HC00268_s_xt	-1.0	1.0	Pyridoxine exchange
EX_Riboflavin	HC00232_s = HC00232_s_xt	-1.0	1.0	Riboflavin exchange
EX_Cholesterol	HC00178_b = HC00178_b_xt	0.0	1.0	Cholesterol export
EX_H2S	HC00250_s = HC00250_s_xt	0.0	1.0	H2S export
EX_Sulfate	HC00062_s = HC00062_s_xt	-1.0	1.0	Sulfate exchange
EX_Urea	HC00089_s = HC00089_s_xt	0.0	1.0	Urea export

Originally sourced from HepatoNet1 PIPES external metabolite exchange set. Modifications were made to represent serum-free, supplemented DMEM nutrient composition. Constraints were set on the metabolite fluxes as measured by [3]. A negative flux represents import while a positive flux represents export, and are set as the lower and upper bounds, respectively. Metabolite fluxes that were not measured by [3] were set accordingly as described by HepatoNet1 PIPES. [1]Metabolites were not measured in [3] and fluxes were set as the lowest minimum and highest maximum flux values from the amino acid flux values from the CORE dataset. The external metabolites are labelled as _xt, the sinusoidal space is _s, and the bile space is _b.
*For qualitative PPARα simulations, all upper and lower bounds were arbitrarily set to range from -1.0 to 1.0 for bidirectional fluxes and 0.0 to 1.0 for unidirectional fluxes.

TABLE S5

Table S5. Generic biomass function used for simulations*

Recon2		HepatoNet1			
CONSUME		ID	Name	Sum formula	Compartments
0.505626 M_ala_L_c	L-alanine	**HC00048**	Alanine	C3H7NO2	c,s,l,m
0.35926 M_arg_L_c	L-arginine	**HC00065**	Arginine	C6H14N4O2	c,s,l,m
0.279425 M_asn_L_c	L-asparagine	**HC00148**	Asparagine	C4H8N2O3	c,s,l,m
0.352607 M_asp_L_c	L-aspartate	**HC00055**	Aspartate	C4H7NO4	c,s,l,m
0.046571 M_cys_L_c	L-cysteine	**HC00099**	Cysteine	C3H7NO2S	c,s,l,m
0.325996 M_gln_L_c	L-glutamine	**HC00067**	Glutamine	C5H10N2O3	c,s,l,m
0.385872 M_glu_L_c	L-glutamate	**HC00034**	Glutamate	C5H9NO4	c,s,l,m
0.538891 M_gly_c	glycine	**HC00045**	Glycine	C2H5NO2	c,s,l,m
0.126406 M_his_L_c	L-histidine	**HC00133**	Histidine	C6H9N3O2	c,s,l,m
0.286078 M_ile_L_c	L-isoleucine	**HC00334**	Isoleucine	C6H13NO2	c,s,l,m
0.545544 M_leu_L_c	L-leucine	**HC00121**	Leucine	C6H13NO2	c,s,l,m
0.592114 M_lys_L_c	L-lysine	**HC00053**	Lysine	C6H14N2O2	c,s,l,m
0.153018 M_met_L_c	L-methionine	**HC00075**	Methionine	C5H11NO2S	c,s,l,m
0.259466 M_phe_L_c	L-phenylalanine	**HC00081**	Phenylalanine	C9H11NO2	c,s,l,m
0.412484 M_pro_L_c	L-proline	**HC00145**	Proline	C5H9NO2	c,s,l,m
0.392525 M_ser_L_c	L-serine	**HC00068**	Serine	C3H7NO3	c,s,l,m
0.31269 M_thr_L_c	L-threonine	**HC00179**	Threonine	C4H9NO3	c,s,l,m
0.013306 M_trp_L_c	L-tryptophan	**HC00080**	Tryptophan	C11H12N2O2	c,s,l
0.159671 M_tyr_L_c	L-tyrosine	**HC00085**	Tyrosine	C9H11NO3	c,s,l,m
0.352607 M_val_L_c	L-valine	**HC00174**	Valine	C5H11NO2	c,s,l,m
0.011658 M_clpn_hs_c	cardiolipin		NOT REPRESENTED IN HEPATONET1		
0.013183 M_datp_n	dATP	**HC00129**	dATP	C10H16N5O12P3	c,m
0.009442 M_dctp_n	dCTP	**HC00367**	dCTP	C9H16N3O13P3	c
0.009898 M_dgtp_n	dGTP	**HC00252**	dGTP	C10H16N5O13P3	c,s,m
0.013091 M_dttp_n	dTTP	**HC00368**	dTTP	C10H17N2O14P3	c,s
0.275194 M_g6p_c	D-glucose-6-phosphate	**HC00094**	Glucose-6P	C6H13O9P	r,c
0.036117 M_gtp_c	GTP	**HC00051**	GTP	C10H16N5O14P3	c,s,m,n
0.053446 M_utp_c	UTP	**HC00077**	UTP	C9H15N2O15P3	c,s
0.039036 M_ctp_c	CTP	**HC00066**	CTP	C9H16N3O14P3	c,s,m,n
20.704451 M_atp_c	ATP	**HC00012**	ATP	C10H16N5O13P3	r,c,s,l,m,p
20.650823 M_h2o_c	H2O	**HC00011**	H2O	H2O	r,c,s,l,m,n,p
0.023315 M_pail_hs_c	phosphatidylinositol	**HC02009**	PI-pool	C11H17O13PR2	r,c,l
0.154463 M_pchol_hs_c	phosphatidylcholine	**HC02080**	Bile-PC-pool	C10H19NO8PR2	b,c
0.055374 M_pe_hs_c	phosphatidylethanolamine	**HC02079**	PE-PS-VLDL-pool	C7H12NO8PR2	c
0.002914 M_pglyc_hs_c	phosphatidylglycerol	**HC02096**	PG-CL-pool	C8H13O10PR2	m
0.005829 M_ps_hs_c	phosphatidylserine	**HC02006**	PS-VLDL-pool	C8H12NO10PR2	r,b,c,l

0.020401 M_chsterol_c	cholesterol	**HC00178**	Cholesterol	C27H46O	r,b,c,s,l
0.017486 M_sphmyln_hs_c	sphingomyelin	**HC02007**	SM-pool	C24H49N2O6PR	r,b,c,l
**PRODUCE**		**ID**	**Name**	**Sum formula**	**Compartments**
20.650823 M_adp_c	ADP	**HC00018**	ADP	C10H15N5O10P2	r,c,s,l,m,p
20.650823 M_h_c	H+	**HC00083**	H+(PG)	H	c,s,m,p
20.650823 M_pi_c	phosphate	**HC00019**	Pi	H3PO4	r,c,s,l,m,p

*generic biomass function adapted from Recon2 [28]

R_biomass_reaction =	0.505626 M_ala_L_c + 0.35926 M_arg_L_c + 0.279425 M_asn_L_c + 0.352607 M_asp_L_c + 20.704451 M_atp_c + 0.020401 M_chsterol_c + 0.011658 M_clpn_hs_c + 0.039036 M_ctp_c + 0.046571 M_cys_L_c + 0.013183 M_datp_n + 0.009442 M_dctp_n + 0.009898 M_dgtp_n + 0.013091 M_dttp_n + 0.275194 M_g6p_c + 0.325996 M_gln_L_c + 0.385872 M_glu_L_c + 0.538891 M_gly_c + 0.036117 M_gtp_c + 20.650823 M_h2o_c + 0.126406 M_his_L_c + 0.286078 M_ile_L_c + 0.545544 M_leu_L_c + 0.592114 M_lys_L_c + 0.153018 M_met_L_c + 0.023315 M_pail_hs_c + 0.154463 M_pchol_hs_c + 0.055374 M_pe_hs_c + 0.002914 M_pglyc_hs_c + 0.259466 M_phe_L_c + 0.412484 M_pro_L_c + 0.005829 M_ps_hs_c + 0.392525 M_ser_L_c + 0.017486 M_sphmyln_hs_c + 0.31269 M_thr_L_c + 0.013306 M_trp_L_c + 0.159671 M_tyr_L_c + 0.053446 M_utp_c + 0.352607 M_val_L_c = 20.650823 M_adp_c + 20.650823 M_h_c + 20.650823 M_pi_c

TABLE S6

Table S6. Custom primer sequences used to quantify PPARα transcript expression via qRT-PCR; primers were synthesised and supplied by Eurofins MWG (Germany).

hPPARα	
Forward Primer	5' – GCA AGA AAT GGG AAA CAT CCA A – 3'
Reverse Primer	5' – TGG TAT TCC GTA AAG CCA AAG CT – 3'
18S	
Forward Primer	5' – CGG CTA CCA CAT CCA AGG AA – 3'
Reverse Primer	5' – GCT GGA ATT ACC GCG GCT – 3'

TABLE S7

Table S7 Target genes and mediated hepatic reactions within PPARα regulome model

Gene*	ENTREZ ID	Protein	Reaction	HepatoNet1 reaction	PMID
**ABAT**	18	4-aminobutyrate aminotransferase	beta-Alanine(m) + AKG(m) <=> Glutamate(m) + 3-Oxopropanoate(m)	r0217	17164430
**ABCA1**	19	ABCA1	Cholesterol(c) + ATP(c) + H2O(c) --> Cholesterol(s) + ADP(c) + Pi(c)<br>Arachidonate(c) + ATP(c) + H2O(c) --> Arachidonate(s) + ADP(c) + Pi(c)<br>Palmitate(c) + ATP(c) + H2O(c) --> Palmitate(s) + ADP(c) + Pi(c)<br>Oleate(c) + ATP(c) + H2O(c) --> Oleate(s) + ADP(c) + Pi(c)<br>Stearate(c) + ATP(c) + H2O(c) --> Stearate(s) + ADP(c) + Pi(c)<br>Linoleate(c) + ATP(c) + H2O(c) --> Linoleate(s) + ADP(c) + Pi(c)<br>Elaidate(c) + ATP(c) + H2O(c) --> Elaidate(s) + ADP(c) + Pi(c)<br>gamma-Linolenate(c) + ATP(c) + H2O(c) --> gamma-Linolenate(s) + ADP(c) +<br>Pi(c)<br>Linolenate(c) + ATP(c) + H2O(c) --> Linolenate(s) + ADP(c) + Pi(c)<br>Lignocerate(c) + ATP(c) + H2O(c) --> Lignocerate(s) + ADP(c) + Pi(c)<br>Palmitolate(c) + ATP(c) + H2O(c) --> Palmitolate(s) + ADP(c) + Pi(c)<br>PC-VLDL-pool(c) + ATP(c) + H2O(c) --> PC-VLDL-pool(s) + ADP(c) + Pi(c)<br>Dihomo-gamma-linolenate(c) + ATP(c) + H2O(c) --> Dihomo-gamma-<br>linolenate(s) + ADP(c) + Pi(c)	r1513<br>r1514<br>r1515<br>r1516<br>r1517<br>r1518<br>r1519<br>r1520<br>r1521<br>r1522<br>r1523<br>r1524<br>r1525<br>r1526<br>r1527	19710929, 18288265, 12512040

*PPARα target gene with no reaction represented within the HepatoNet1 GSMN

			GM4-pool(c) + ATP(c) + H2O(c) --> GM4-pool(s) + ADP(c) + Pi(c)<br>decanoic-acid(c) + ATP(c) + H2O(c) --> decanoic-acid(s) + ADP(c) + Pi(c)<br>lauric-acid(c) + ATP(c) + H2O(c) --> lauric-acid(s) + ADP(c) + Pi(c)<br>myristic-acid(c) + ATP(c) + H2O(c) --> myristic-acid(s) + ADP(c) + Pi(c)<br>ApoA1(c) <=> ApoA1(s)	r1528<br>r1529<br>r0808	
**ABCB4**	5244	MDR3	Cholesterol(c) + ATP(c) + H2O(c) --> Cholesterol(b) + ADP(c) + Pi(c)<br>PE-VLDL-pool(c) + ATP(c) + H2O(c) --> PE-VLDL-pool(b) + ADP(c) + Pi(c)<br>SM-pool(c) + ATP(c) + H2O(c) --> SM-pool(b) + ADP(c) + Pi(c)<br>Bile-PC-pool(c) + ATP(c) + H2O(c) --> Bile-PC-pool(b) + ADP(c) + Pi(c)	r1508<br>r1509<br>r1510<br>r1511	19710929, 18288265, 12512040, 12381268
**ABCD2**	225	ATP-binding cassette sub-family D member 2	Arachidonate(c) + ATP(c) + H2O(c) --> Arachidonate(p) + ADP(c) + Pi(c)<br>Palmitate(c) + ATP(c) + H2O(c) --> Palmitate(p) + ADP(c) + Pi(c)<br>Propanoyl-CoA(c) + ATP(c) + H2O(c) --> Propanoyl-CoA(p) + ADP(c) + Pi(c)<br>Palmitoyl-CoA(c) + ATP(c) + H2O(c) --> Palmitoyl-CoA(p) + ADP(c) + Pi(c)<br>Choloyl-CoA(c) + ATP(c) + H2O(c) --> Choloyl-CoA(p) + ADP(c) + Pi(c)<br>Linoleoyl-CoA(c) + ATP(c) + H2O(c) --> Linoleoyl-CoA(p) + ADP(c) + Pi(c)<br>Arachidonyl-CoA(c) + ATP(c) + H2O(c) --> Arachidonyl-CoA(p) + ADP(c) + Pi(c)	r2497<br>r2498<br>r2499<br>r2500<br>r2501<br>r2502<br>r2503	18288265, 11422379
**ABCD3**	5825	ATP-binding cassette sub-family D member 3	half transporter - functional heterodimer with D2	-	18288265, 11422379
**ACAA1A/ ACAA2**	30/10449	3-ketoacyl-CoA thiolase	Propanoyl-CoA(m) + Acetyl-CoA(m) <=> CoA(m) + 2-Methylacetoacetyl-CoA(m)<br>Acetyl-CoA(m) + Butyryl-CoA(m) <-- CoA(m) + 3-Oxohexanoyl-CoA(m)	r0222<br>r0287<br>r0628	18288265, 12946649, 12729718

			Propanoyl-CoA(p) + Choloyl-CoA(p) <-- CoA(p) + 3alpha,7alpha,12alpha-Trihydroxy-5beta-24-oxocholestanoyl-CoA(p)<br>Acetyl-CoA(m) + Octanoyl-CoA(m) <=> CoA(m) + 3-Oxodecanoyl-CoA(m)<br>Acetyl-CoA(p) + Octanoyl-CoA(p) <-- CoA(p) + 3-Oxodecanoyl-CoA(p)<br>Lauroyl-CoA(m) + Acetyl-CoA(m) <-- CoA(m) + 3-Oxotetradecanoyl-CoA(m)<br>Lauroyl-CoA(p) + Acetyl-CoA(p) <-- CoA(p) + 3-Oxotetradecanoyl-CoA(p)<br>Myristoyl-CoA(m) + Acetyl-CoA(m) <-- CoA(m) + 3-Oxopalmitoyl-CoA(m)<br>Myristoyl-CoA(p) + Acetyl-CoA(p) <-- CoA(p) + 3-Oxopalmitoyl-CoA(p)<br>Propanoyl-CoA(p) + Chenodeoxycholoyl-CoA(p) <=> CoA(p) + 3alpha,7alpha-Dihydroxy-5beta-cholestanoyl-CoA(p) + H2O(p)<br>Decanoyl-CoA(m) + Acetyl-CoA(m) <=> CoA(m) + 3-Oxododecanoyl-CoA(m)<br>Decanoyl-CoA(p) + Acetyl-CoA(p) <-- CoA(p) + 3-Oxododecanoyl-CoA(p)<br>Hexanoyl-CoA(m) + Acetyl-CoA(m) <-- CoA(m) + 3-Oxooctanoyl-CoA(m)	r0634<br>r0635<br>r0639<br>r0640<br>r0653<br>r0654<br>r0698<br>r0724<br>r0725<br>r0732	
**ACACB**	32	Acetyl-CoA carboxylase 2	ATP(c) + Acetyl-CoA(c) + HCO3-(c) --> ADP(c) + Pi(c) + Malonyl-CoA(c)<br>ATP(m) + Acetyl-CoA(m) + HCO3-(m) --> ADP(m) + Pi(m) + Malonyl-CoA(m)	r0184<br>r0185	18288265
**ACADM**	34	Medium-chain specific acyl-CoA dehydrogenase, mitochondrial	Palmitoyl-CoA(m) + Ubiquinone(m) --> (2E)-Hexadecenoyl-CoA(m) + Ubiquinol(m)<br>Propanoyl-CoA(m) + Ubiquinone(m) --> Ubiquinol(m) + Acrylyl-CoA(m)	r0310<br>r0683	10377439, 18288265, 9488698
**ACADS**	35	Short-chain specific acyl-CoA dehydrogenase, mitochondrial	Isobutyryl-CoA(m) + Ubiquinone(m) --> Methacrylyl-CoA(m) + Ubiquinol(m)	r0560	10359558, 18288265, 9488698

**ACADVL**	37	Very long-chain specific acyl-CoA dehydrogenase, mitochondrial	Palmitoyl-CoA(m) + Ubiquinone(m) --> (2E)-Hexadecenoyl-CoA(m) + Ubiquinol(m)<br>Propanoyl-CoA(m) + Ubiquinone(m) --> Ubiquinol(m) + Acrylyl-CoA(m)	r0310<br>r0683	19710929, 18288265, 17118139, 9488698
**ACOT12**	134526	Acyl-coenzyme A thioesterase 12	Acetyl-CoA(c) + H2O(c) --> CoA(c) + Acetate(c)<br>Acetyl-CoA(m) + H2O(m) --> CoA(m) + Acetate(m)	r0061<br>r0062	18288265
**ACOX1**	51	Peroxisomal acyl-coenzyme A oxidase 1	O2(p) + 3alpha,7alpha,12alpha-Trihydroxy-5beta-cholestanoyl-CoA(p) --> 3alpha,7alpha,12alpha-Trihydroxy-5beta-cholest-24-enoyl-CoA(p) + H2O2(p)<br>Ubiquinone(m) + Butyryl-CoA(m) --> Crotonyl-CoA(m) + Ubiquinol(m)<br>Ubiquinone(m) + Lauroyl-CoA(m) --> (2E)-Dodecenoyl-CoA(m) + Ubiquinol(m)<br>Ubiquinone(m) + Octanoyl-CoA(m) --> (2E)-Octenoyl-CoA(m) + Ubiquinol(m)<br>Ubiquinone(m) + Myristoyl-CoA(m) --> (2E)-Tetradecenoyl-CoA(m) + Ubiquinol(m)<br>Ubiquinone(m) + Hexanoyl-CoA(m) --> (2E)-Hexenoyl-CoA(m) + Ubiquinol(m)<br>Ubiquinone(m) + Decanoyl-CoA(m) --> (2E)-Decenoyl-CoA(m) + Ubiquinol(m)<br>Palmitoyl-CoA(m) + Ubiquinone(m) --> (2E)-Hexadecenoyl-CoA(m) + Ubiquinol(m)<br>Propanoyl-CoA(m) + Ubiquinone(m) --> Ubiquinol(m) + Acrylyl-CoA(m)	r0706<br>r1446<br>r1447<br>r1448<br>r1449<br>r1450<br>r1451<br>r0310<br>r0683	19710929, 10377439, 18288265,
**ACSL1**	2180	Long chain fatty acid CoA ligase 1	Palmitate(r) + ATP(r) + CoA(r) --> Palmitoyl-CoA(r) + AMP(r) + PPi(r)<br>Palmitate(c) + ATP(c) + CoA(c) --> Palmitoyl-CoA(c) + AMP(c) + PPi(c)<br>Palmitolate(r) + ATP(r) + CoA(r) --> (2E)-Hexadecenoyl-CoA(r) + AMP(r) + PPi(r)<br>Palmitolate(c) + ATP(c) + CoA(c) --> (2E)-Hexadecenoyl-CoA(c) + AMP(c) + PPi©	r0311<br>r0312<br>r1251<br>r1252<br>r1253<br>r1254	19710929, 18288265, 11371553, 7642600, 17118139

			Stearate(r) + ATP(r) + CoA(r) --> Stearoyl-CoA(r) + AMP(r) + PPi(r)<br>Stearate(c) + ATP(c) + CoA(c) --> Stearoyl-CoA(c) + AMP(c) + PPi(c)<br>ATP(r) + CoA(r) + Oleate(r) --> Oleoyl-CoA(r) + AMP(r) + PPi(r)<br>ATP(c) + CoA(c) + Oleate(c) --> Oleoyl-CoA(c) + AMP(c) + PPi(c)<br>Linoleate(r) + ATP(r) + CoA(r) --> AMP(r) + PPi(r) + Linoleoyl-CoA(r)<br>Linoleate(c) + ATP(c) + CoA(c) --> AMP(c) + PPi(c) + Linoleoyl-CoA(c)<br>ATP(r) + CoA(r) + Linolenate(r) --> Linolenoyl-CoA(r) + AMP(r) + PPi(r)<br>gamma-Linolenate(r) + ATP(r) + CoA(r) --> gamma-Linolenoyl-CoA(r) + AMP(r) + PPi(r)<br>gamma-Linolenate(c) + ATP(c) + CoA(c) --> gamma-Linolenoyl-CoA(c) + AMP(c) + PPi(c)<br>Arachidonate(r) + ATP(r) + CoA(r) --> Arachidonyl-CoA(r) + AMP(r) + PPi(r)<br>Arachidonate(c) + ATP(c) + CoA(c) --> Arachidonyl-CoA(c) + AMP(c) + PPi(c)<br>ATP(r) + CoA(r) + Palmitolate(r) <=> AMP(r) + palmitoleoyl-CoA(r) + PPi(r)<br>ATP(c) + CoA(c) + Palmitolate(c) <=> AMP(c) + palmitoleoyl-CoA(c) + PPi(c)	r1255<br>r1256<br>r1257<br>r1258<br>r1259<br>r1260<br>r1261<br>r1262<br>r1263<br>r1487<br>r1488	
**ACSL3**	2181	Long chain fatty acid CoA ligase 3	Palmitate(r) + ATP(r) + CoA(r) --> Palmitoyl-CoA(r) + AMP(r) + PPi(r)<br>Palmitate(c) + ATP(c) + CoA(c) --> Palmitoyl-CoA(c) + AMP(c) + PPi(c)<br>Palmitolate(r) + ATP(r) + CoA(r) --> (2E)-Hexadecenoyl-CoA(r) + AMP(r) + PPi(r)<br>Palmitolate(c) + ATP(c) + CoA(c) --> (2E)-Hexadecenoyl-CoA(c) + AMP(c) + PPi©<br>Stearate(r) + ATP(r) + CoA(r) --> Stearoyl-CoA(r) + AMP(r) + PPi(r)<br>Stearate(c) + ATP(c) + CoA(c) --> Stearoyl-CoA(c) + AMP(c) + PPi(c)<br>ATP(r) + CoA(r) + Oleate(r) --> Oleoyl-CoA(r) + AMP(r) + PPi(r)	r0311<br>r0312<br>r1251<br>r1252<br>r1253<br>r1254<br>r1255<br>r1256<br>r1257	19710929, 18288265

			ATP(c) + CoA(c) + Oleate(c) --> Oleoyl-CoA(c) + AMP(c) + PPi(c)<br>Linoleate(r) + ATP(r) + CoA(r) --> AMP(r) + PPi(r) + Linoleoyl-CoA(r)<br>Linoleate(c) + ATP(c) + CoA(c) --> AMP(c) + PPi(c) + Linoleoyl-CoA(c)<br>ATP(r) + CoA(r) + Linolenate(r) --> Linolenoyl-CoA(r) + AMP(r) + PPi(r)<br>gamma-Linolenate(r) + ATP(r) + CoA(r) --> gamma-Linolenoyl-CoA(r) + AMP(r) + PPi(r)<br>gamma-Linolenate(c) + ATP(c) + CoA(c) --> gamma-Linolenoyl-CoA(c) + AMP(c) + PPi(c)<br>Arachidonate(r) + ATP(r) + CoA(r) --> Arachidonyl-CoA(r) + AMP(r) + PPi(r)<br>Arachidonate(c) + ATP(c) + CoA(c) --> Arachidonyl-CoA(c) + AMP(c) + PPi(c)<br>ATP(r) + CoA(r) + Palmitolate(r) <=> AMP(r) + palmitoleoyl-CoA(r) + PPi(r)<br>ATP(c) + CoA(c) + Palmitolate(c) <=> AMP(c) + palmitoleoyl-CoA(c) + PPi(c)	r1258<br>r1259<br>r1260<br>r1261<br>r1262<br>r1263<br>r1487<br>r1488	
**ACSL5**	51703	Long chain fatty acid CoA ligase 5	Palmitate(r) + ATP(r) + CoA(r) --> Palmitoyl-CoA(r) + AMP(r) + PPi(r)<br>Palmitate(c) + ATP(c) + CoA(c) --> Palmitoyl-CoA(c) + AMP(c) + PPi(c)<br>Palmitolate(r) + ATP(r) + CoA(r) --> (2E)-Hexadecenoyl-CoA(r) + AMP(r) + PPi(r)<br>Palmitolate(c) + ATP(c) + CoA(c) --> (2E)-Hexadecenoyl-CoA(c) + AMP(c) + PPi©<br>Stearate(r) + ATP(r) + CoA(r) --> Stearoyl-CoA(r) + AMP(r) + PPi(r)<br>Stearate(c) + ATP(c) + CoA(c) --> Stearoyl-CoA(c) + AMP(c) + PPi(c)<br>ATP(r) + CoA(r) + Oleate(r) --> Oleoyl-CoA(r) + AMP(r) + PPi(r)<br>ATP(c) + CoA(c) + Oleate(c) --> Oleoyl-CoA(c) + AMP(c) + PPi(c)<br>Linoleate(r) + ATP(r) + CoA(r) --> AMP(r) + PPi(r) + Linoleoyl-CoA(r)<br>Linoleate(c) + ATP(c) + CoA(c) --> AMP(c) + PPi(c) + Linoleoyl-CoA(c)	r0311<br>r0312<br>r1251<br>r1252<br>r1253<br>r1254<br>r1255<br>r1256<br>r1257<br>r1258<br>r1259<br>r1260	19710929, 18288265, 17118139

			ATP(r) + CoA(r) + Linolenate(r) --> Linolenoyl-CoA(r) + AMP(r) + PPi(r)<br>gamma-Linolenate(r) + ATP(r) + CoA(r) --> gamma-Linolenoyl-CoA(r) + AMP(r) + PPi(r)<br>gamma-Linolenate(c) + ATP(c) + CoA(c) --> gamma-Linolenoyl-CoA(c) + AMP(c) + PPi(c)<br>Arachidonate(r) + ATP(r) + CoA(r) --> Arachidonyl-CoA(r) + AMP(r) + PPi(r)<br>Arachidonate(c) + ATP(c) + CoA(c) --> Arachidonyl-CoA(c) + AMP(c) + PPi(c)<br>ATP(r) + CoA(r) + Palmitolate(r) <=> AMP(r) + palmitoleoyl-CoA(r) + PPi(r)<br>ATP(c) + CoA(c) + Palmitolate(c) <=> AMP(c) + palmitoleoyl-CoA(c) + PPi(c)	r1261<br>r1262<br>r1263<br>r1487<br>r1488	
**ACSM3***	6296	Acyl-coenzyme A synthetase, mitochondial	-	-	24269660
**AGPAT2**	10555	1-acyl-sn-glycerol-3-phosphate acyltransferase beta	Phosphatidate-VLDL-TG-pool(c) + CoA(c) <-- Acyl-CoA-VLDL-TG2-pool(c) + 1-Acylglycerol-3P-VLDL-TG1-pool(c)<br>Phosphatidate-VLDL-PC-pool(c) + CoA(c) <-- Acyl-CoA-VLDL-PC-pool(c) + 1-Acylglycerol-3P-VLDL-PC-pool(c)<br>Phosphatidate-VLDL-PE-pool(c) + CoA(c) <-- Acyl-CoA-VLDL-PE-pool(c) + 1-Acylglycerol-3P-VLDL-PE-pool(c)<br>Phosphatidate-VLDL-PS-pool(c) + CoA(c) <-- Acyl-CoA-VLDL-PS-pool(c) + 1-Acylglycerol-3P-VLDL-PS-pool(c)<br>Phosphatidate-VLDL-PI-pool(c) + CoA(c) <-- 1-Acylglycerol-3P-VLDL-PI-pool(c) + Acyl-CoA-VLDL-PI-pool(c)<br>CoA(c) + Phosphatidate-VLDL-SM-pool(c) <-- Acyl-CoA-VLDL-SM-pool(c) + 1-Acylglycerol-3P-VLDL-SM-pool(c)	r1211<br>r1212<br>r1213<br>r1214<br>r1215<br>r1216<br>r1286<br>r1308	18288265

			Phosphatidate-Bile-PC-pool(c) + CoA(c) <-- Acyl-CoA-Bile-PC-pool(c) + 1-Acylglycerol-3P-Bile-PC-pool(c)<br>Phosphatidate-CL-pool(m) + CoA(m) <-- Acyl-CoA-CL-pool(m) + 1-Acylglycerol-3P-CL-pool(m)		
**AGXT2**	64902	Alanine-glyoxylate aminotransferase 2, mitochondrial	D-3-Amino-isobutanoate(m) + Pyruvate(m) <=> 2-Methyl-3-oxopropanoate(m) + Alanine(m)	r0484	24269660
**AKR1B10**	57016	Aldo-keto reductase family member B10	Glycerol(c) + NADP+(c) <=> Glyceraldehyde(c) + NADPH(c)<br>Glycerol(m) + NADP+(m) --> Glyceraldehyde(m) + NADPH(m)	r0245<br>r0246	19710929
**AKR1C3***	8644	Aldo-keto reductase family member c3	-	-	24269660
**ALAS1**	211	5-aminolevulinate synthase, nonspecific, mitochondrial	Glycine(m) + Succinyl-CoA(m) <=> CoA(m) + CO2(m) + 5-Aminolevulinate(m)<br>Glycine(m) + Succinyl-CoA(m) <=> CoA(m) + 2-Amino-3-oxoadipate(m)<br>2-Amino-3-oxoadipate(m) <=> CO2(m) + 5-Aminolevulinate(m)	r0195<br>r0196<br>r0517	24269660
**ALDH3A1**	218	Aldehyde dehydrogenase, dimeric NADP-preferring	Acetaldehyde(c) + NAD+(c) + H2O(c) --> Acetate(c) + NADH(c)<br>Acetaldehyde(c) + H2O(c) + NADP+(c) --> Acetate(c) + NADPH(c)<br>NAD+(c) + H2O(c) + Phenylacetaldehyde(c) <=> NADH(c) + Phenylacetate(c)<br>NAD+(m) + H2O(m) + Phenylacetaldehyde(m) <=> NADH(m) + Phenylacetate(m)	r0176<br>r0177<br>r0545<br>r0546<br>r0547<br>r0548	19710929

			H2O(c) + Phenylacetaldehyde(c) + NADP+(c) <=> NADPH(c) + Phenylacetate(c)<br>H2O(m) + Phenylacetaldehyde(m) + NADP+(m) <=> NADPH(m) + Phenylacetate(m)<br>3,4-Dihydroxymandelaldehyde(m) + NAD+(m) + H2O(m) <=> 3,4-Dihydroxymandelate(m) + NADH(m)<br>3,4-Dihydroxymandelaldehyde(m) + H2O(m) + NADP+(m) <=> 3,4-Dihydroxymandelate(m) + NADPH(m)<br>3-Methoxy-4-hydroxyphenylacetaldehyde(m) + NAD+(m) + H2O(m) <=> NADH(m) + Homovanillate(m)<br>3-Methoxy-4-hydroxyphenylacetaldehyde(m) + H2O(m) + NADP+(m) <=> NADPH(m) + Homovanillate(m)<br>3-Methoxy-4-hydroxyphenylglycolaldehyde(m) + NAD+(m) + H2O(m) <=> NADH(m) + Vanillylmandelate(m)<br>3-Methoxy-4-hydroxyphenylglycolaldehyde(m) + H2O(m) + NADP+(m) <=> NADPH(m) + Vanillylmandelate(m)	r0752<br>r0753<br>r0754<br>r0755<br>r0756<br>r0757	
**ALDH3A2**	224	Fatty aldehyde dehydrogenase	Acetaldehyde(c) + NAD+(c) + H2O(c) --> Acetate(c) + NADH(c)<br>Glyceraldehyde(c) + NAD+(c) + H2O(c) <=> Glycerate(c) + NADH(c)<br>Glyceraldehyde(m) + NAD+(m) + H2O(m) --> Glycerate(m) + NADH(m)<br>H2O(m) + 4-Aminobutanal(m) + NADP+(m) <=> 4-Aminobutyrate(m) + NADPH(m)<br>NAD+(m) + H2O(m) + 4-Aminobutanal(m) <=> 4-Aminobutyrate(m) + NADH(m)	r0176<br>r0392<br>r0393<br>r0464<br>r0549<br>r0642<br>r0643<br>r0688	19710929, 18288265, 20032461

			2-Methyl-3-oxopropanoate(c) + NAD+(c) + H2O(c) <=> Methylmalonate(c) + NADH(c)<br>2-Methyl-3-oxopropanoate(m) + NAD+(m) + H2O(m) <=> Methylmalonate(m) + NADH(m)<br>3alpha,7alpha-Dihydroxy-5beta-cholestan-26-al(c) + NAD+(c) + H2O(c) <=> NADH(c) + 3alpha,7alpha-Dihydroxy-5beta-cholestanate(c)<br>5-Hydroxyindoleacetaldehyde(c) + NAD+(c) + H2O(c) <=> NADH(c) + 5-Hydroxyindoleacetate(c)	r0758	
**ALDH9A1**	223	TMABADH	Acetaldehyde(c) + NAD+(c) + H2O(c) --> Acetate(c) + NADH(c)<br>Glyceraldehyde(c) + NAD+(c) + H2O(c) <=> Glycerate(c) + NADH(c)<br>Glyceraldehyde(m) + NAD+(m) + H2O(m) --> Glycerate(m) + NADH(m)<br>H2O(m) + 4-Aminobutanal(m) + NADP+(m) <=> 4-Aminobutyrate(m) + NADPH(m)<br>NAD+(m) + H2O(m) + 4-Aminobutanal(m) <=> 4-Aminobutyrate(m) + NADH(m)<br>2-Methyl-3-oxopropanoate(c) + NAD+(c) + H2O(c) <=> Methylmalonate(c) + NADH(c)<br>2-Methyl-3-oxopropanoate(m) + NAD+(m) + H2O(m) <=> Methylmalonate(m) + NADH(m)<br>3alpha,7alpha-Dihydroxy-5beta-cholestan-26-al(c) + NAD+(c) + H2O(c) <=> NADH(c) + 3alpha,7alpha-Dihydroxy-5beta-cholestanate(c)<br>5-Hydroxyindoleacetaldehyde(c) + NAD+(c) + H2O(c) <=> NADH(c) + 5-Hydroxyindoleacetate(c)	r0176<br>r0392<br>r0393<br>r0464<br>r0549<br>r0642<br>r0643<br>r0688<br>r0758	19710929

**ANGPTL 4***	51129	Angiopoietin-related protein 4	-	-	19710929, 18288265, 15190076
**APOA1**	335	Apolipoprotein A-I	23 Alanine(c) + 17 Arginine(c) + 19 Glutamine(c) + 30 Glutamate(c) + 11 Glycine(c) + 6 Histidine(c) + 41 Leucine(c) + 4 Methionine(c) + 8 Phenylalanine(c) + 10 Proline(c) + 16 Serine(c) + 12 Threonine(c) + 5 Tryptophan(c) + 7 Tyrosine(c) + 15 Valine(c) + 1068 ATP(c) + 1068 H2O(c) + 5 Asparagine(c) + 16 Aspartate(c) + 22 Lysine(c) --> ApoA1(c) + 267 AMP(c) + 267 PPi(c) + 801 ADP(c) + 801 Pi(c)	r1113	8647932, 9748239, 7983038
**APOA2***	336	Apolipoprotein A-II	-	-	19710929, 7635967
**APOA5***	116519	Apolipoprotein A-V	-	-	19710929, 12709436, 12637506
**APOCIII**	345	Apolipoprotein C-III	15 Alanine(c) + 4 Arginine(c) + 3 Glycine(c) + Histidine(c) + 11 Leucine(c) + 3 Methionine(c) + 4 Phenylalanine(c) + 3 Proline(c) + 12 Serine(c) + 5 Threonine(c) + 3 Tryptophan(c) + 2 Tyrosine(c) + 9 Valine(c) + 6 Glutamine(c) + 5 Glutamate(c) + 396 ATP(c) + 396 H2O(c) + 7 Aspartate(c) + 6 Lysine(c) --> ApoC3(c) + 99 AMP(c) + 99 PPi(c) + 297 ADP(c) + 297 Pi(c)	r1100	7768950, 7860752, 8847480
**AQP3**	360	Aquaporin-3	H2O(s) <=> H2O(c)	r0849	18288265, 15232616
**ASL**	435	Argininosuccinate lyase	Argininosuccinate(c) <=> Fumarate(c) + Arginine(c)	r0261	17164430, 20847941

**CBS**	875	Cystathionine beta-synthase	Serine(c) + H2S(c) --> Cysteine(c) + H2O(c)<br>Serine(c) + Homocysteine(c) <=> H2O(c) + L-Cystathionine(c)	r0210<br>r0314	17164430
**CPS-1**	1373	Carbamoyl-phosphate synthase (ammonia), mitochondrial	2 ATP(m) + CO2(m) + H2O(m) + NH3(m) <=> 2 ADP(m) + Pi(m) + Carbamoyl-P(m)	r0034	24269660
**CPT1A/CPT2**	1374/1376	CPT	Malonyl-CoA(r) + L-Carnitine(r) <=> CoA(r) + Malonyl-Carnitin(r)<br>Malonyl-CoA(c) + L-Carnitine(c) <=> CoA(c) + Malonyl-Carnitin(c)<br>Malonyl-CoA(m) + L-Carnitine(m) <=> CoA(m) + Malonyl-Carnitin(m)<br>Malonyl-CoA(p) + L-Carnitine(p) <-- CoA(p) + Malonyl-Carnitin(p)<br>Palmitoyl-CoA(r) + L-Carnitine(r) <=> CoA(r) + L-Palmitoylcarnitine(r)<br>Palmitoyl-CoA(c) + L-Carnitine(c) <=> CoA(c) + L-Palmitoylcarnitine(c)<br>Palmitoyl-CoA(m) + L-Carnitine(m) <-- CoA(m) + L-Palmitoylcarnitine(m)<br>Palmitoyl-CoA(p) + L-Carnitine(p) <=> CoA(p) + L-Palmitoylcarnitine(p)<br>Linoleoyl-CoA(r) + L-Carnitine(r) <=> CoA(r) + linoleic-Carnitine(r)<br>Linoleoyl-CoA(c) + L-Carnitine(c) <=> CoA(c) + linoleic-Carnitine(c)<br>Linoleoyl-CoA(p) + L-Carnitine(p) --> CoA(p) + linoleic-Carnitine(p)<br>Arachidonyl-CoA(r) + L-Carnitine(r) <=> CoA(r) + Arachidonyl-Carnitine(r)<br>Arachidonyl-CoA(c) + L-Carnitine(c) <=> CoA(c) + Arachidonyl-Carnitine(c)<br>Arachidonyl-CoA(p) + L-Carnitine(p) --> CoA(p) + Arachidonyl-Carnitine(p)<br>palmitoleoyl-CoA(r) + L-Carnitine(r) <=> CoA(r) + palmitoleoyl-Carnitine(r)<br>palmitoleoyl-CoA(c) + L-Carnitine(c) <=> CoA(c) + palmitoleoyl-Carnitine(c)<br>palmitoleoyl-CoA(m) + L-Carnitine(m) <=> CoA(m) + palmitoleoyl-Carnitine(m)	r0430<br>r0431<br>r0432<br>r0433<br>r0434<br>r0435<br>r0436<br>r0437<br>r0438<br>r0439<br>r0440<br>r0441<br>r0442<br>r0443<br>r0444<br>r0445<br>r0446	19710929, 10359558, 10377439, 18288265, 9726988, 9535828, 9488698, 12408750

			Oleoyl-CoA(c) + L-Carnitine(c) --> CoA(c) + L-Oleoylcarnitine(c)<br>Propanoyl-CoA(c) + L-Carnitine(c) --> CoA(c) + O-Propanoylcarnitine(c)<br>Butyryl-CoA(c) + L-Carnitine(c) --> CoA(c) + O-Butanoylcarnitine(c)	r1394<br>r1396<br>r1398	
**CYP1A2***	1544	Cytochrome P450 1A2	-	-	24269660
**CYP3A5***	1577	Cytochrome P450 3A5	-	-	24269660
**CYP3A7***	1551	Cytochrome P450 3A7	-	-	24269660
**CYP7A1**	1581	Cholesterol 7 alpha monooxygenase	Cholesterol(r) + O2(r) + NADPH(r) <=> 7alpha-Hydroxycholesterol(r) + H2O(r) + NADP+(r)	r0335	11701475, 11042130, 10777541
**CYP27A1**	1593	Sterol 27-hydroxylase, mitochondrial	5beta-Cholestane-3alpha,7alpha-diol(m) + NADPH(m) + O2(m) <=> 5beta-Cholestane-3alpha,7alpha,26-triol(m) + H2O(m) + NADP+(m)<br>3alpha,7alpha,12alpha-Trihydroxycoprostane(c) + NADPH(c) + O2(c) <=> 5beta-Cholestane-3alpha,7alpha,12alpha,26-tetrol(c) + H2O(c) + NADP+(c)	r0740<br>r0741<br>r0742	11701475, 24269660

			3alpha,7alpha,12alpha-Trihydroxycoprostane(m) + NADPH(m) + O2(m) <=> 5beta-Cholestane-3alpha,7alpha,12alpha,26-tetrol(m) + H2O(m) + NADP+(m)		
**CYP2B6***	1555	Cytochrome P450 2B6	-	-	24269660
**CYP8B1**	1582	7-alpha-hydroxycholest-4-en-3-one 12-alpha-hydroxylase	7alpha-Hydroxycholest-4-en-3-one(r) + O2(r) + NADPH(r) --> 7alpha,12alpha-Dihydroxycholest-4-en-3-one(r) + H2O(r) + NADP+(r)	r0751	10867000, 24269660
**CYP2C8***	1558	Cytochrome P450 2C8	-	-	19710929, 24269660
**CYP2C9***	1559	Cytochrome P450 2C9	-	-	19710929, 24269660
**CYP2J2***	1573	Cytochrome P450 2J2	-	-	19710929, 24269660
**ELOVL5**	60481	Elongation of very long chain fatty acids protein 5	Hexadecanoyl-ACP(c) + Malonyl-ACP(c) <=> 3-Oxostearoyl-ACP(c) + ACP(c) + CO2(c)	r1313	18288265, 16790840, 15654130
**ELOVL6**	79071	Elongation of very long chain fatty acids protein 6	Hexadecanoyl-ACP(c) + Malonyl-ACP(c) <=> 3-Oxostearoyl-ACP(c) + ACP(c) + CO2(c)	r1313	18288265, 16790840, 15654130
**EPHX2***	2053	Bifunctional epoxide hyrolase 2	-	-	24269660

**ETFDH***	2110	Electron transfer flavoprotein-ubiquinone oxidoreductase, mitochodrial	-	-	19710929
**FABP1**	2168	Fatty acid-binding protein, liver	Arachidonate(l) --> Arachidonate(r)<br>Arachidonate(l) <=> Arachidonate(c)<br>Palmitate(l) --> Palmitate(r)<br>Palmitate(l) <=> Palmitate(c)<br>Stearate(l) --> Stearate(r)<br>Stearate(l) <=> Stearate(c)	r0931<br>r0932<br>r0936<br>r0937<br>r0983<br>r0984	19710929, 18288265, 10383430, 11284737
**FADS1***	3992	Fatty acid desaturase 1	-	-	18288265, 16790840
**FADS2***	9415	Fatty acid desaturase 2	-	-	18288265, 16790840, 12562861
**FGF21***	26291	Fibroblast growth factor 21	-	-	19710929, 17550777, 17550778
**G6PC**	2538	Glucose-6-phophatase	H2O(r) + Glucose-6P(r) --> Glucose(r) + Pi(r)	r0396	19710929
**GK**	2710	Glycerol kinase	ATP(c) + Glycerol(c) <=> ADP(c) + sn-Glycerol-3P(c)<br>ATP(m) + Glycerol(m) <=> ADP(m) + sn-Glycerol-3P(m)	r0203<br>r0204	24269660

**GLS2**	27165	Glutaminase liver isoform, mitochondrial	Glutamine(m) + H2O(m) --> Glutamate(m) + NH3(m)	r0078	17164430
**GPAM**	57678	Glycerol-3-phosphate acyltransferase 1, mitochondrial	Palmitoyl-CoA(c) + sn-Glycerol-3P(c) --> CoA(c) + 1-Acylglycerol-3P-palm(c)<br>(2E)-Hexadecenoyl-CoA(c) + sn-Glycerol-3P(c) --> 1-Acylglycerol-3P-palmn(c) + CoA(c)<br>Stearoyl-CoA(c) + sn-Glycerol-3P(c) --> CoA(c) + 1-Acylglycerol-3P-stea(c)<br>Oleoyl-CoA(c) + sn-Glycerol-3P(c) --> 1-Acylglycerol-3P-ol(c) + CoA(c)<br>Linoleoyl-CoA(c) + sn-Glycerol-3P(c) --> 1-Acylglycerol-3P-lin(c) + CoA(c)<br>Arachidonyl-CoA(c) + sn-Glycerol-3P(c) --> 1-Acylglycerol-3P-arach(c) + CoA(c)<br>Acyl-CoA-CL-pool(m) + sn-Glycerol-3P(m) --> CoA(m) + 1-Acylglycerol-3P-CL-pool(m)	r1185<br>r1186<br>r1187<br>r1188<br>r1189<br>r1190<br>r1307	19710929, 18288265
**GPT**	2875	Alanine aminotransferase 1	Alanine(c) + AKG(c) <=> Pyruvate(c) + Glutamate(c)<br>Alanine(m) + AKG(m) <=> Pyruvate(m) + Glutamate(m)	r0080<br>r0081	24269660
**HACL1***	26061	2-hydroxyacyl-CoA lyase 1	-	-	19710929
**HADHA**	3030	Trifunctional enzyme subunit alpha, mitochondrial	(S)-3-Hydroxybutyryl-CoA(m) <=> Crotonyl-CoA(m) + H2O(m)<br>3-Hydroxypropionyl-CoA(m) <-- H2O(m) + Acrylyl-CoA(m)<br>(S)-3-Hydroxydodecanoyl-CoA(m) <=> (2E)-Dodecenoyl-CoA(m) + H2O(m)<br>(S)-3-Hydroxydodecanoyl-CoA(p) <-- (2E)-Dodecenoyl-CoA(p) + H2O(p)<br>(S)-3-Hydroxy-2-methylbutyryl-CoA(m) <=> H2O(m) + Tiglyl-CoA(m)<br>Methacrylyl-CoA(m) + H2O(m) --> 3-Hydroxyisobutyryl-CoA(m)	r0588<br>r0592<br>r0660<br>r0661<br>r0665<br>r0669	19710929, 18288265, 17118139, 16197558

			(S)-3-Hydroxyhexadecanoyl-CoA(m) <-- (2E)-Hexadecenoyl-CoA(m) + H2O(m)<br>(S)-3-Hydroxyhexadecanoyl-CoA(p) <-- (2E)-Hexadecenoyl-CoA(p) + H2O(p)<br>(S)-3-Hydroxytetradecanoyl-CoA(m) <-- (2E)-Tetradecenoyl-CoA(m) + H2O(m)<br>(S)-3-Hydroxytetradecanoyl-CoA(p) <-- (2E)-Tetradecenoyl-CoA(p) + H2O(p)<br>(S)-Hydroxydecanoyl-CoA(m) <=> (2E)-Decenoyl-CoA(m) + H2O(m)<br>(S)-Hydroxydecanoyl-CoA(p) <-- (2E)-Decenoyl-CoA(p) + H2O(p)<br>(S)-Hydroxyoctanoyl-CoA(m) <-- (2E)-Octenoyl-CoA(m) + H2O(m)<br>(S)-Hydroxyhexanoyl-CoA(m) <-- (2E)-Hexenoyl-CoA(m) + H2O(m)<br>NAD+(m) + (S)-3-Hydroxyhexadecanoyl-CoA(m) --> 3-Oxopalmitoyl-CoA(m) + NADH(m)<br>NAD+(p) + (S)-3-Hydroxyhexadecanoyl-CoA(p) --> 3-Oxopalmitoyl-CoA(p) + NADH(p)<br>(S)-3-Hydroxytetradecanoyl-CoA(m) + NAD+(m) --> 3-Oxotetradecanoyl-CoA(m) + NADH(m)<br>(S)-3-Hydroxytetradecanoyl-CoA(p) + NAD+(p) --> 3-Oxotetradecanoyl-CoA(p) + NADH(p)<br>(S)-3-Hydroxydodecanoyl-CoA(m) + NAD+(m) <=> 3-Oxododecanoyl-CoA(m) + NADH(m)<br>(S)-3-Hydroxydodecanoyl-CoA(p) + NAD+(p) --> 3-Oxododecanoyl-CoA(p) + NADH(p)<br>(S)-Hydroxydecanoyl-CoA(m) + NAD+(m) <=> 3-Oxodecanoyl-CoA(m) + NADH(m)	r0716<br>r0717<br>r0720<br>r0721<br>r0728<br>r0729<br>r0731<br>r0734<br>r0714<br>r0715<br>r0718<br>r0719<br>r0722<br>r0723<br>r0726<br>r0727<br>r0730<br>r0733	

			(S)-Hydroxydecanoyl-CoA(p) + NAD+(p) --> 3-Oxodecanoyl-CoA(p) + NADH(p)<br>(S)-Hydroxyoctanoyl-CoA(m) + NAD+(m) --> 3-Oxooctanoyl-CoA(m) + NADH(m)<br>(S)-Hydroxyhexanoyl-CoA(m) + NAD+(m) --> 3-Oxohexanoyl-CoA(m) + NADH(m)		
**HADHB**	3032	Trifunctional enzyme subunit beta, mitochondrial	Propanoyl-CoA(m) + Acetyl-CoA(m) <=> CoA(m) + 2-Methylacetoacetyl-CoA(m)<br>Acetyl-CoA(m) + Butyryl-CoA(m) <-- CoA(m) + 3-Oxohexanoyl-CoA(m)<br>Propanoyl-CoA(p) + Choloyl-CoA(p) <-- CoA(p) + 3alpha,7alpha,12alpha-Trihydroxy-5beta-24-oxocholestanoyl-CoA(p)<br>Acetyl-CoA(m) + Octanoyl-CoA(m) <=> CoA(m) + 3-Oxodecanoyl-CoA(m)<br>Acetyl-CoA(p) + Octanoyl-CoA(p) <-- CoA(p) + 3-Oxodecanoyl-CoA(p)<br>Lauroyl-CoA(m) + Acetyl-CoA(m) <-- CoA(m) + 3-Oxotetradecanoyl-CoA(m)<br>Lauroyl-CoA(p) + Acetyl-CoA(p) <-- CoA(p) + 3-Oxotetradecanoyl-CoA(p)<br>Myristoyl-CoA(m) + Acetyl-CoA(m) <-- CoA(m) + 3-Oxopalmitoyl-CoA(m)<br>Myristoyl-CoA(p) + Acetyl-CoA(p) <-- CoA(p) + 3-Oxopalmitoyl-CoA(p)<br>Propanoyl-CoA(p) + Chenodeoxycholoyl-CoA(p) <=> CoA(p) + 3alpha,7alpha-Dihydroxy-5beta-cholestanoyl-CoA(p) + H2O(p)<br>Decanoyl-CoA(m) + Acetyl-CoA(m) <=> CoA(m) + 3-Oxododecanoyl-CoA(m)<br>Decanoyl-CoA(p) + Acetyl-CoA(p) <-- CoA(p) + 3-Oxododecanoyl-CoA(p)<br>Hexanoyl-CoA(m) + Acetyl-CoA(m) <-- CoA(m) + 3-Oxooctanoyl-CoA(m)	r0222<br>r0287<br>r0628<br>r0634<br>r0635<br>r0639<br>r0640<br>r0653<br>r0654<br>r0698<br>r0724<br>r0725<br>r0732	19710929,<br>18288265,<br>17118139

**HMGCS1**	3157	HMG CoA synthase, cytoplasmic	HMG-CoA(c) + CoA(c) <=> Acetyl-CoA(c) + H2O(c) + Acetoacetyl-CoA(c)<br>HMG-CoA(m) + CoA(m) <=> Acetyl-CoA(m) + H2O(m) + Acetoacetyl-CoA(m)<br>HMG-CoA(p) + CoA(p) <=> Acetyl-CoA(p) + H2O(p) + Acetoacetyl-CoA(p)	r0461<br>r0462<br>r0463	18288265,
**HMGCS2**	3158	HMG CoA synthase, mitochondrial	HMG-CoA(c) + CoA(c) <=> Acetyl-CoA(c) + H2O(c) + Acetoacetyl-CoA(c)<br>HMG-CoA(m) + CoA(m) <=> Acetyl-CoA(m) + H2O(m) + Acetoacetyl-CoA(m)<br>HMG-CoA(p) + CoA(p) <=> Acetyl-CoA(p) + H2O(p) + Acetoacetyl-CoA(p)	r0461<br>r0462<br>r0463	19710929, 18288265, 10869548, 12381268, 7913466
**HSD17B4**	3295	Peroxisomal multifunctional enzyme type 2	3alpha,7alpha,12alpha,24-Tetrahydroxy-5beta-cholestanoyl-CoA(p) <-- 3alpha,7alpha,12alpha-Trihydroxy-5beta-cholest-24-enoyl-CoA(p) + H2O(p)<br>(S)-3-Hydroxybutyryl-CoA(m) + NAD+(m) <=> NADH(m) + Acetoacetyl-CoA(m)<br>NAD+(m) + (S)-3-Hydroxyhexadecanoyl-CoA(m) --> 3-Oxopalmitoyl-CoA(m) + NADH(m)<br>NAD+(p) + (S)-3-Hydroxyhexadecanoyl-CoA(p) --> 3-Oxopalmitoyl-CoA(p) + NADH(p)<br>(S)-3-Hydroxytetradecanoyl-CoA(m) + NAD+(m) --> 3-Oxotetradecanoyl-CoA(m) + NADH(m)<br>(S)-3-Hydroxytetradecanoyl-CoA(p) + NAD+(p) --> 3-Oxotetradecanoyl-CoA(p) + NADH(p)<br>(S)-3-Hydroxydodecanoyl-CoA(m) + NAD+(m) <=> 3-Oxododecanoyl-CoA(m) + NADH(m)<br>(S)-3-Hydroxydodecanoyl-CoA(p) + NAD+(p) --> 3-Oxododecanoyl-CoA(p) + NADH(p)	r0744<br>r0460<br>r0714<br>r0715<br>r0718<br>r0719<br>r0722<br>r0723<br>r0726<br>r0727<br>r0733<br>r0743	18288265, 9771468

			(S)-Hydroxydecanoyl-CoA(m) + NAD+(m) <=> 3-Oxodecanoyl-CoA(m) + NADH(m)<br>(S)-Hydroxydecanoyl-CoA(p) + NAD+(p) --> 3-Oxodecanoyl-CoA(p) + NADH(p)<br>(S)-Hydroxyhexanoyl-CoA(m) + NAD+(m) --> 3-Oxohexanoyl-CoA(m) + NADH(m)<br>3alpha,7alpha,12alpha,24-Tetrahydroxy-5beta-cholestanoyl-CoA(p) + NAD+(p) --> 3alpha,7alpha,12alpha-Trihydroxy-5beta-24-oxocholestanoyl-CoA(p) + NADH(p)		
**LEPR**	3953	Leptin receptor	-	-	18288265
**LIPC**	3990	HTGL	Triacylglycerol-VLDL-pool(c) + H2O(c) --> Fatty-acid-VLDL-TG3-pool(c) + 1,2-Diacylglycerol-VLDL-TG-pool(c)<br>H2O(c) + 1,2-Diacylglycerol-VLDL-TG-pool(c) --> 1-Acylglycerol-VLDL-TG1-pool(c) + Fatty-acid-VLDL-TG2-pool(c)<br>H2O(c) + 1,2-Diacylglycerol-VLDL-PC-pool(c) --> 1-Acylglycerol-VLDL-PC-pool(c) + Fatty-acid-VLDL-PC-pool(c)<br>H2O(c) + 1,2-Diacylglycerol-VLDL-PE-pool(c) --> 1-Acylglycerol-VLDL-PE-pool(c) + Fatty-acid-VLDL-PE-pool(c)<br>H2O(c) + 1,2-Diacylglycerol-VLDL-PS-pool(c) --> 1-Acylglycerol-VLDL-PS-pool(c) + Fatty-acid-VLDL-PS-pool(c)<br>H2O(c) + 1,2-Diacylglycerol-VLDL-PI-pool(c) --> 1-Acylglycerol-VLDL-PI-pool(c) + Fatty-acid-VLDL-PI-pool(c)<br>H2O(c) + 1,2-Diacylglycerol-VLDL-SM-pool(c) --> 1-Acylglycerol-VLDL-SM-pool(c) + Fatty-acid-VLDL-SM-pool(c)	r1224<br>r1225<br>r1226<br>r1227<br>r1228<br>r1229<br>r1230	24269660

**ME1**	4199	NADP-dependent malic enzyme	Malate(c) + NADP+(c) <=> Pyruvate(c) + CO2(c) + NADPH(c)<br>Malate(m) + NADP+(m) <=> Pyruvate(m) + CO2(m) + NADPH(m)	r0058<br>r0059	24269660
**MGST3***	4259	Microsomal GST-3	-	-	19710929
**NR1H3***	10062	LXR	-	-	12512040, 10809236
**NR1H4***	9971	FXR	-	-	18288265
**OAT**	4942	Ornithine aminotransferase	AKG(m) + Ornithine(m) <=> L-Glutamate_5-semialdehyde(m) + Glutamate(m)	r0167	17164430
**ODC1**	4953	Ornithine decarboxylase	Ornithine(c) <=> Putrescine(c) + CO2(c)	r0168	17164430
**OTC**	5009	Ornithine carbmoyltransferase, mitochondrial	Carbamoyl-P(m) + Ornithine(m) <=> Pi(m) + Citrulline(m)	r0329	24269660
**PAH**	5053	Phenylalanine-4-hydroxylase	Tetrahydrobiopterin(c) + Phenylalanine(c) + O2(c) <=> Dihydrobiopterin(c) + Tyrosine(c) + H2O(c)	r0399	17164430
**PCK1**	5105	Phosphoenolpyruvate carboxykinase, cytosolic	GTP(c) + OAA(c) --> GDP(c) + PEP(c) + CO2(c)	r0123	19710929
**PDK4***	5166	Pyruvate dehyrogenase (acetyl-transferring)	-	-	19710929, 18288265, 12023878

		kinase isozyme 4, mitochondrial			
**PCTP***	58488	Phosphatidylcholine transfer protein	-	-	19710929, 18288265
**PLTP***	5360	Phospholipid transfer protein	-	-	18288265, 11342537
**PSAT1**	29968	Phosphoserine aminotransferase	3-Phosphoserine(c) + AKG(c) <-- 3-Phosphonooxypyruvate(c) + Glutamate(c)	r0663	17164430
**SCD**	6319	Acyl-CoA desaturase	Palmitoyl-CoA(c) + O2(c) + 2 Ferrocytochrome_b5(c) <=> palmitoleoyl-CoA(c) + 2 Ferricytochrome_b5(c) + 2 H2O(c)<br>Stearoyl-CoA(c) + O2(c) + 2 Ferrocytochrome_b5(c) <=> Oleoyl-CoA(c) + 2 Ferricytochrome_b5(c) + 2 H2O(c)<br>2 H2O(c) + NAD+(c) + Oleoyl-CoA(c) <=> NADH(c) + O2(c) + Stearoyl-CoA(c)	r0510<br>r0511<br>r1465	18288265, 8790349
**SCP2**	6342	Non-specific lipid-transfer protein	Propanoyl-CoA(p) + Choloyl-CoA(p) <-- CoA(p) + 3alpha,7alpha,12alpha-Trihydroxy-5beta-24-oxocholestanoyl-CoA(p)	r0628	24269660
**SLC22A5**	6584	Solute carrier family 22 member 5	Histamine(s) <=> Histamine(c)<br>Dopamine(s) <=> Dopamine(c)<br>Lysine(c) <=> Lysine(s)<br>Methionine(s) <=> Methionine(c)	r0952<br>r1009<br>r1069<br>r1072	19710929, 18288265, 17692817
**SLC25A20**	788	Mitochondrial carnitine/acylcarnitine carrier protein	L-Carnitine(c) + O-Acetylcarnitine(r) <=> L-Carnitine(r) + O-Acetylcarnitine(c)<br>L-Carnitine(m) + O-Acetylcarnitine(c) --> L-Carnitine(c) + O-Acetylcarnitine(m)<br>L-Carnitine(m) + L-Octanoylcarnitine(c) --> L-Carnitine(c) + L-Octanoylcarnitine(m)	r0995<br>r2433<br>r2434<br>r2435	19710929, 18288265

			L-Carnitine(m) + L-Palmitoylcarnitine(c) --> L-Carnitine(c) + L-Palmitoylcarnitine(m)<br>L-Carnitine(m) + L-Oleoylcarnitine(c) --> L-Carnitine(c) + L-Oleoylcarnitine(m)<br>L-Carnitine(m) + O-Propanoylcarnitine(c) --> L-Carnitine(c) + O-Propanoylcarnitine(m)<br>L-Carnitine(m) + O-Butanoylcarnitine(c) --> L-Carnitine(c) + O-Butanoylcarnitine(m)	r2436<br>r2437<br>r2438	
**SLC27A2**	11001	Very long-chain acyl- CoA synthetase (aka Fatty acid transport protein 2, FATP2)	Arachidonate(s) --> Arachidonate(c)<br>Palmitate(s) --> Palmitate(c)<br>Stearate(s) --> Stearate(c)<br>Stearate(l) --> Stearate(r)<br>Stearate(l) <=> Stearate(c)<br>Linoleate(s) --> Linoleate(c)<br>gamma-Linolenate(s) --> gamma-Linolenate(c)<br>Oleate(s) --> Oleate(c)<br>Linolenate(s) --> Linolenate(c)<br>Dihomo-gamma-linolenate(s) --> Dihomo-gamma-linolenate(c)<br>Elaidate(s) --> Elaidate(c)<br>Lignocerate(s) --> Lignocerate(c)<br>Palmitolate(s) --> Palmitolate(c)<br>myristic-acid(s) --> myristic-acid(c)<br>Palmitate(r) + ATP(r) + CoA(r) --> Palmitoyl-CoA(r) + AMP(r) + PPi(r)<br>Palmitate(c) + ATP(c) + CoA(c) --> Palmitoyl-CoA(c) + AMP(c) + PPi(c)	r0930<br>r0935<br>r0982<br>r0983<br>r0984<br>r0985<br>r1297<br>r1300<br>r1363<br>r1366<br>r2440<br>r2441<br>r2442<br>r2445<br>r0311<br>r0312<br>r1251	18288265, 17118139

			Palmitolate(r) + ATP(r) + CoA(r) --> (2E)-Hexadecenoyl-CoA(r) + AMP(r) + PPi(r)<br>Palmitolate(c) + ATP(c) + CoA(c) --> (2E)-Hexadecenoyl-CoA(c) + AMP(c) + PPi(c)<br>Stearate(r) + ATP(r) + CoA(r) --> Stearoyl-CoA(r) + AMP(r) + PPi(r)<br>Stearate(c) + ATP(c) + CoA(c) --> Stearoyl-CoA(c) + AMP(c) + PPi(c)<br>ATP(r) + CoA(r) + Oleate(r) --> Oleoyl-CoA(r) + AMP(r) + PPi(r)<br>ATP(c) + CoA(c) + Oleate(c) --> Oleoyl-CoA(c) + AMP(c) + PPi(c)<br>Linoleate(r) + ATP(r) + CoA(r) --> AMP(r) + PPi(r) + Linoleoyl-CoA(r)<br>Linoleate(c) + ATP(c) + CoA(c) --> AMP(c) + PPi(c) + Linoleoyl-CoA(c)<br>ATP(r) + CoA(r) + Linolenate(r) --> Linolenoyl-CoA(r) + AMP(r) + PPi(r)<br>gamma-Linolenate(r) + ATP(r) + CoA(r) --> gamma-Linolenoyl-CoA(r) + AMP(r) + PPi(r)<br>gamma-Linolenate(c) + ATP(c) + CoA(c) --> gamma-Linolenoyl-CoA(c) + AMP(c) + PPi(c)<br>Arachidonate(r) + ATP(r) + CoA(r) --> Arachidonyl-CoA(r) + AMP(r) + PPi(r)<br>Arachidonate(c) + ATP(c) + CoA(c) --> Arachidonyl-CoA(c) + AMP(c) + PPi(c)<br>ATP(r) + CoA(r) + Palmitolate(r) <=> AMP(r) + palmitoleoyl-CoA(r) + PPi(r)<br>ATP(c) + CoA(c) + Palmitolate(c) <=> AMP(c) + palmitoleoyl-CoA(c) + PPi(c)	r1252<br>r1253<br>r1254<br>r1255<br>r1256<br>r1257<br>r1258<br>r1259<br>r1260<br>r1261<br>r1262<br>r1263<br>r1487<br>r1488	

			Arachidonate(s) --> Arachidonate(c)	r0930	
			Palmitate(s) --> Palmitate(c)	r0935	
			Stearate(s) --> Stearate(c)	r0982	
			Stearate(l) --> Stearate(r)	r0983	
			Stearate(l) <=> Stearate(c)	r0984	
			Linoleate(s) --> Linoleate(c)	r0985	
			gamma-Linolenate(s) --> gamma-Linolenate(c)	r1297	
			Oleate(s) --> Oleate(c)	r1300	
			Linolenate(s) --> Linolenate(c)	r1363	
			Dihomo-gamma-linolenate(s) --> Dihomo-gamma-linolenate(c)	r1366	
		Long-chain fatty	Elaidate(s) --> Elaidate(c)	r2440	
**SLC27A4**	10999	acid transport	Palmitolate(s) --> Palmitolate(c)	r2442	19710929,
		protein	myristic-acid(s) --> myristic-acid(c)	r2445	18288265
			Palmitate(r) + ATP(r) + CoA(r) --> Palmitoyl-CoA(r) + AMP(r) + PPi(r)	r0311	
			Palmitate(c) + ATP(c) + CoA(c) --> Palmitoyl-CoA(c) + AMP(c) + PPi(c)	r0312	
			Palmitolate(r) + ATP(r) + CoA(r) --> (2E)-Hexadecenoyl-CoA(r) + AMP(r) +	r1251	
			PPi(r)	r1252	
			Palmitolate(c) + ATP(c) + CoA(c) --> (2E)-Hexadecenoyl-CoA(c) + AMP(c) +	r1253	
			PPi©	r1254	
			Stearate(r) + ATP(r) + CoA(r) --> Stearoyl-CoA(r) + AMP(r) + PPi(r)	r1255	
			Stearate(c) + ATP(c) + CoA(c) --> Stearoyl-CoA(c) + AMP(c) + PPi(c)	r1256	
			ATP(r) + CoA(r) + Oleate(r) --> Oleoyl-CoA(r) + AMP(r) + PPi(r)	r1257	
			ATP(c) + CoA(c) + Oleate(c) --> Oleoyl-CoA(c) + AMP(c) + PPi(c)	r1258	
			Linoleate(r) + ATP(r) + CoA(r) --> AMP(r) + PPi(r) + Linoleoyl-CoA(r)	r1259	

			Linoleate(c) + ATP(c) + CoA(c) --> AMP(c) + PPi(c) + Linoleoyl-CoA(c)<br>ATP(r) + CoA(r) + Linolenate(r) --> Linolenoyl-CoA(r) + AMP(r) + PPi(r)<br>gamma-Linolenate(r) + ATP(r) + CoA(r) --> gamma-Linolenoyl-CoA(r) + AMP(r) + PPi(r)<br>gamma-Linolenate(c) + ATP(c) + CoA(c) --> gamma-Linolenoyl-CoA(c) + AMP(c) + PPi(c)<br>Arachidonate(r) + ATP(r) + CoA(r) --> Arachidonyl-CoA(r) + AMP(r) + PPi(r)<br>Arachidonate(c) + ATP(c) + CoA(c) --> Arachidonyl-CoA(c) + AMP(c) + PPi(c)<br>ATP(r) + CoA(r) + Palmitolate(r) <=> AMP(r) + palmitoleoyl-CoA(r) + PPi(r)<br>ATP(c) + CoA(c) + Palmitolate(c) <=> AMP(c) + palmitoleoyl-CoA(c) + PPi(c)	r1260<br>r1261<br>r1262<br>r1263<br>r1487<br>r1488	
**TXNIP***	10628	Thioredoxin-interacting protein	-	-	19710929, 18288265
**UGT1A9**	54600	UDPGT 1-9	Bilirubin(r) + 2 UDP-glucuronate(r) --> 2 UDP(r) + Bilirubin-bisglucuronoside(r)	r0532	24269660
**VLDLR**	7436	Very low density lipoprotein receptor	VLDL(s) --> VLDL(l)	r1084	19710929, 18288265

*PPARα target gene with no reaction represented within the HepatoNet1 GSMN

REFERENCES

1. Kubota H *et al.* Temporal Coding of Insulin Action through Multiplexing of the AKT Pathway. *Mol Cell* **46** 820-832 (2012).
2. Gille C *et al.* HepatoNet1: a comprehensive metabolic reconstruction of the human hepatocyte for the analysis of liver physiology. *Mol Syst Biol* **6** 1-13 (2010).
3. Jain M *et al.* Metabolite Profiling Identifies a Key Role for Glycine in Rapid Cancer Cell Proliferation. *Science* **336** 1040-1044 (2012).
4. Heiner M, Herajy M, Liu F, Rohr C, and Schwarick M. Snoopy - A unifying Petri net tool. *Lecture Notes in Computer Science* **7347 LNCS** 398-407 (2012).
5. Fisher CP, Plant NJ, Moore JB, and Kierzek AM. QSSPN: dynamic simulation of molecular interaction networks describing gene regulation, signalling and whole-cell metabolism in human cells. *Bioinformatics* **29** 1-9 (2013).
6. Wu H *et al.* MUFINS : Multi-Formalism Interaction Network Simulator. *NPJ Systems Biol Appl* **2** 16032 (2016).
7. Baur H and Heldt HW. Transport of hexoses across the liver-cell membrane. *Eur J Biochem* **74** 397-403 (1977).
8. Lemonnier F, Feneant M, Moatti N, Gautier M, and Lemonnier A. D-glucose uptake in human liver cell cultures. *In Vitro* **17** 745-751 (1981).
9. Elliott KR and Craik JD. Sugar transport across the hepatocyte plasma membrane. *Biochem Soc Transact* **10** 12-13 (1982).
10. Mardones L *et al.* The glucose transporter-2 (GLUT2) is a low affinity dehydroascorbic acid transporter. *Biochem Biophys Res Comm* **410** 7-12 (2011).
11. Bell GI, Burant CF, Takeda J, and Gould GW. Structure and function of mammalian facilitative sugar transporters. *J Biol Chem* **268** 19161-19164 (1993).
12. Takanaga H, Chaudhuri B, and Frommer W. GLUT1 and GLUT9 as major contributors to glucose influx in HepG2 cells identified by a high sensitivity intramolecular FRET glucose sensor. *Biochimica et Biophysica acta* **1778** 1091-1099 (2008).
13. Guo L, Dial S, Shi L, Branham W, and Liu J. Similarities and differences in the expression of drug-metabolizing enzymes between human hepatic cell lines and primary human hepatocytes. *Drug Metabol Disposit* **39** 528-538 (2011).
14. Hirahatake K, Meissen J, Fiehn O, and Adams S. Comparative effects of fructose and glucose on lipogenic gene expression and intermediary metabolism in HepG2 liver cells. *PLoS ONE* **6** 1-9 (2011).
15. Levi J *et al.* Fluorescent fructose derivatives for imaging breast cancer cells. *Bioconjugate Chem* **18** 628-634 (2007).
16. Mueckler M and Thorens B. The SLC2 (GLUT) family of membrane transporters. *Mol Aspects Med* **34** 121-138 (2013).
17. Augustin R and Mayoux E. Mammalian Sugar Transporters. *Glucose Homeostasis*: InTech; 2014. pp. 3-36.
18. Li C *et al.* BioModels Database: An enhanced, curated and annotated resource for published quantitative kinetic models. *BMC Systems Biology* **4** 92 (2010).
19. Hoops S *et al.* COPASI - A COmplex PAthway SImulator. *Bioinformatics* **22** 3067-3074 (2006).
20. Peak M, Rochford JJ, Borthwick AC, Yeaman SJ, and Agius L. Signalling pathways involved in the stimulation of glycogen synthesis by insulin in rat hepatocytes. *Diabetologia* **41** 16-25 (1998).
21. Mostafalou S *et al.* Molecular mechanisms involved in lead induced disruption of hepatic and pancreatic glucose metabolism. *Environ Toxicol Pharmacol* **39** 16-26 (2015).

22. Barthel A and Schmoll D. Novel concepts in insulin regulation of hepatic gluconeogenesis. *Am J Physiol Endocrinol Metab* **285** E685-692 (2003).
23. Juty N *et al.* BioModels: Content, features, functionality, and use. *CPT Pharmacometrics Syst Pharmacol* **4** 55-68 (2015).
24. Lanaspa M *et al.* Endogenous fructose production and metabolism in the liver contributes to the development of metabolic syndrome. *Nat Commun* **4** 2434 (2013).
25. Mayes PA. Intermediary metabolism of fructose. *Am J Clin Nutr* **58** 754S-765S (1993).
26. Uhlen M *et al.* Tissue-based map of the human proteome. *Science* **347** 1260419-1260419 (2015).
27. Tazawa S *et al.* SLC5A9/SGLT4, a new Na +-dependent glucose transporter, is an essential transporter for mannose, 1,5-anhydro-D-glucitol, and fructose. *Life Sciences* **76** 1039-1050 (2005).
28. Thiele I *et al.* A community-driven global reconstruction of human metabolism. *Nat Biotechnol* **31** 419-425 (2013).
29. Nyberg SL *et al.* Primary hepatocytes outperform Hep G2 cells as the source of biotransformation functions in a bioartificial liver. *Annals Surg* **220** 59-67 (1994).
30. Kicic A, Chua ACG, and Baker E. Desferrithiocin is a more potent antineoplastic agent than desferrioxamine. *Brit J Pharmacol* **135** 1393-1402 (2002).
31. Hermes M, von Hippel S, Osswald H, and Kloor D. S-adenosylhomocysteine metabolism in different cell lines: effect of hypoxia and cell density. *Cell Physiol Biochem* **15** 233-244 (2005).
32. Norouzzadeh M, Kalikias Y, Mohamadpur Z, and Sharifi L. Determining population doubling time and the appropriate number of HepG2 cells for culturing in 6- well plate. *Int Res J Basic Appl Sci* **10** 299-303 (2016).
33. Stocchi V, Magnani M, Canestrari F, Dachà M, and Fornaini G. Multiple forms of human red blood cell hexokinase. Preparation, characterization, and age dependence. *J Biol Chem* **257** 2357-2364 (1982).
34. Tanimoto T, Ohta M, Tanaka A, Ikemoto I, and Machida T. Purification and characterization of human testis aldose and aldehyde reductase. *Int J Biochem* **23** 421-428 (1991).
35. Sato S, Old S, Carper D, and Kador PF. Purification and characterization of recombinant human placental and rat lens aldose reductases expressed in Escherichia coli. *Adv Exp Med Biol* **372** 259-268 (1995).
36. Brown KE *et al.* Immunodetection of aldose reductase in normal and diseased human liver. *Histol Histopathol* **20** 429-436 (2005).
37. Maldonado EM *et al.* Integration of Genome Scale Metabolic Networks and gene regulation of metabolic enzymes with Physiologically Based Pharmacokinetics. *PT Pharmacometrics Syst Pharmacol* (2017).
38. Mahadevan R and Schilling CH. The effects of alternate optimal solutions in constraint-based genome-scale metabolic models. *Metabol Eng* **5** 264-276 (2003).
39. Kanehisa M, Furumichi M, Tanabe M, Sato Y, and Morishima K. KEGG: new perspectives on genomes, pathways, diseases and drugs. *Nuc Acids Res* **45** D353-D361 (2017).
40. Nishimura D. BioCarta. *Biotech Software and Internet Report* **2** 117-120 (2001).
41. Huang da W, Sherman BT, and Lempicki RA. Systematic and integrative analysis of large gene lists using DAVID bioinformatics resources. *Nat Protoc* **4** 44-57 (2009).
42. Krzywinski M, Birol I, Jones SJM, and Marra MA. Hive plots—rational approach to visualizing networks. *Briefings in Bioinformatics* **13** 627-644 (2012).
43. Levy KJ, Mazzatti DJ, and Moore JB. Gene expression analyses of palmitate-induced steatosis in human hepatocytes. *Proc Nutr Soc* **69** (2010).
44. Spanos C *et al.* Proteomic characterization of dysregulated hepatic glyoxalase 1 in non-alcoholic fatty liver disease. *Proteome Sci.* **16**:4 (2018)

45. Lodish H, Berk A, and Zipursky S. The Molecules of Life. *Molecular Cell Biology*. 4th ed. New York: W. H. Freeman and Company; 2000.
46. Niklas J, Noor F, and Heinzle E. Effects of drugs in subtoxic concentrations on the metabolic fluxes in human hepatoma cell line Hep G2. *Toxicol Appl Pharmacol* **240** 327-336 (2009).
